# Supplementary material for: Dynamic interaction of REEP5–MFN1/2 enables mitochondrial hitchhiking on tubular ER
Source: J Cell Biol. 2024 Aug 12;223(10):e202304031. doi: 10.1083/jcb.202304031 (PMC11318672; doi:10.1083/jcb.202304031)

Fig. 1A

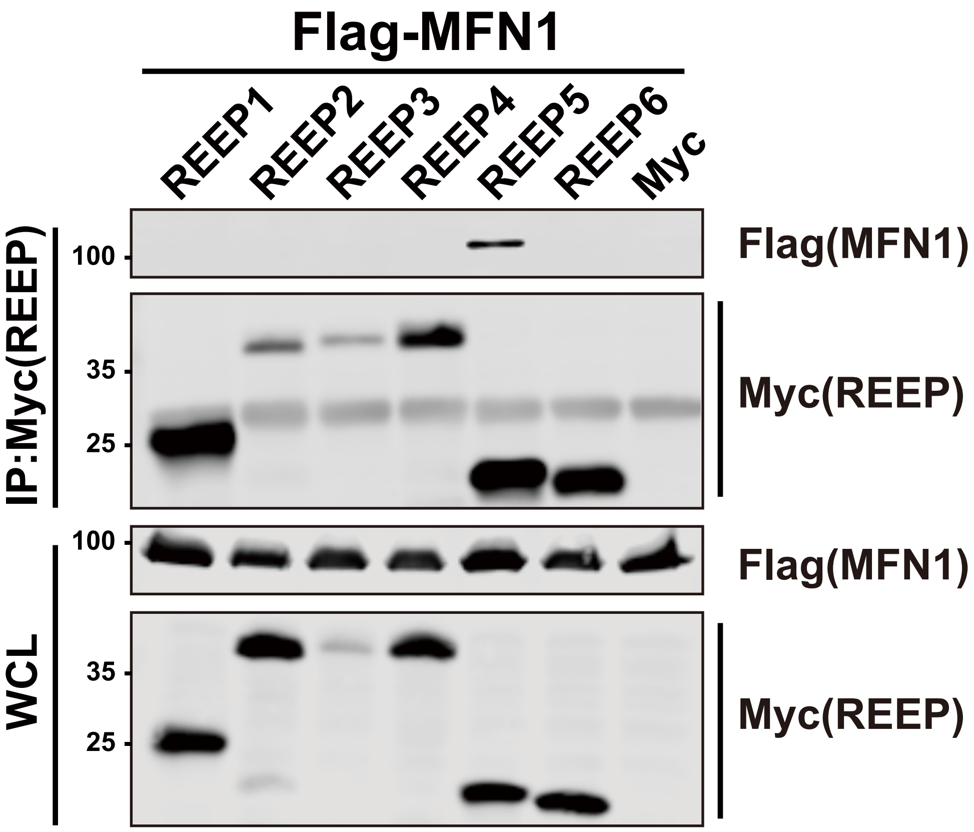
Flag-MFN1
REEP1
REEP2
REEP3
REEP4
REEP5
REEP6
Myc
IP:Myc(REEP)
100
35
25
Flag(MFN1)
Myc(REEP)
WCL
100
35
25
Flag(MFN1)
Myc(REEP)

Source data for Fig. 1A

Orginal image files from which Fig. 1A was assembled.

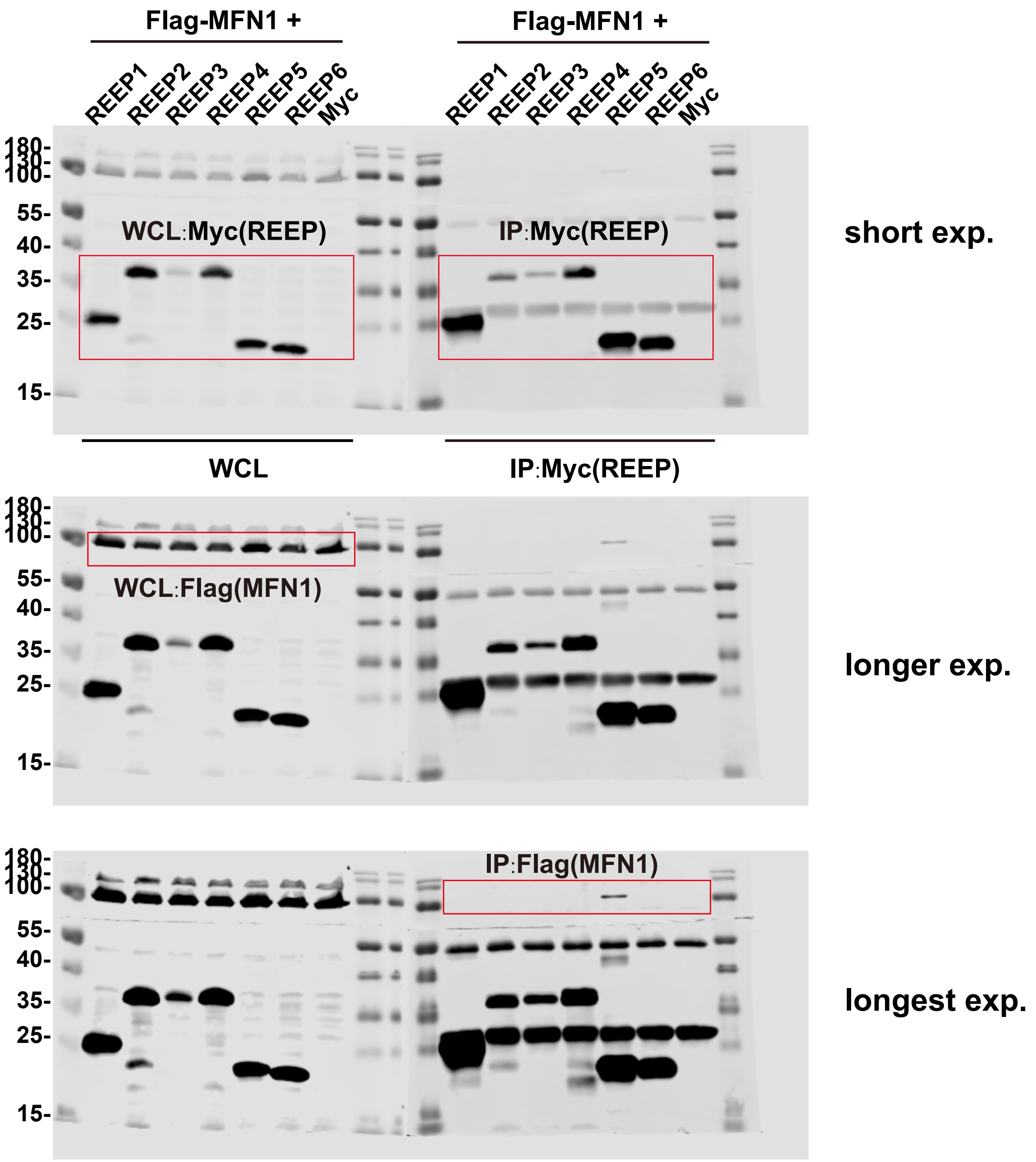

Fig. 1B

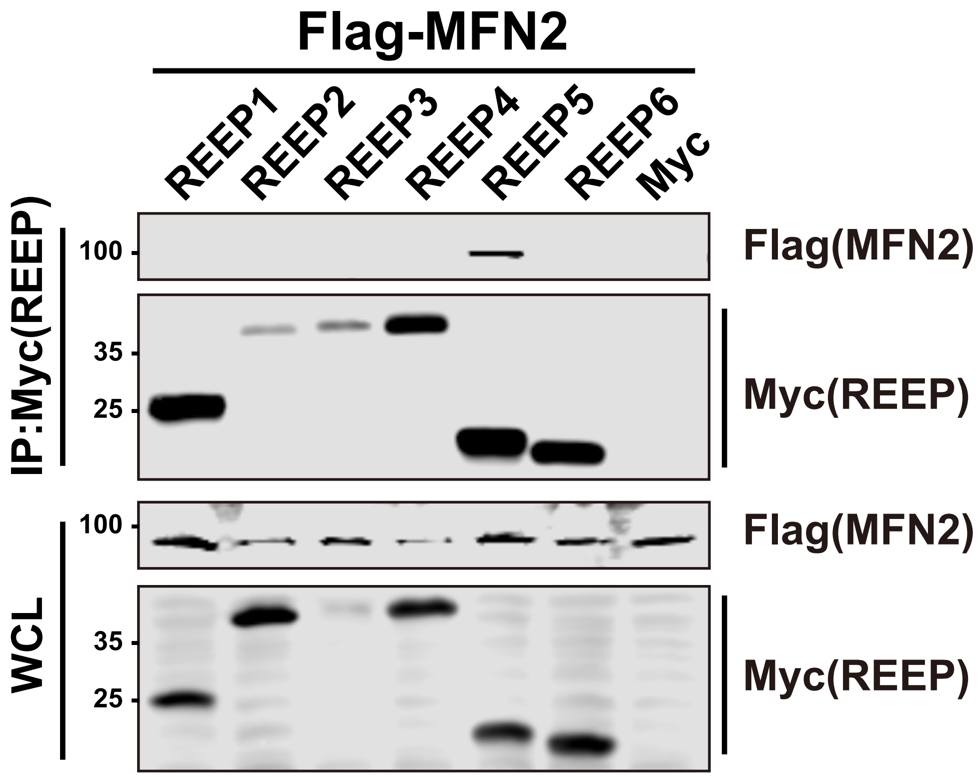

Flag-MFN2
REEP1
REEP2
REEP3
REEP4
REEP5
REEP6
Myc
IP:Myc(REEP)
100
35
25
Flag(MFN2)
Myc(REEP)
WCL
100
35
25
Flag(MFN2)
Myc(REEP)

Source data for Fig. 1B

Orginal image files from which Fig. 1B was assembled.

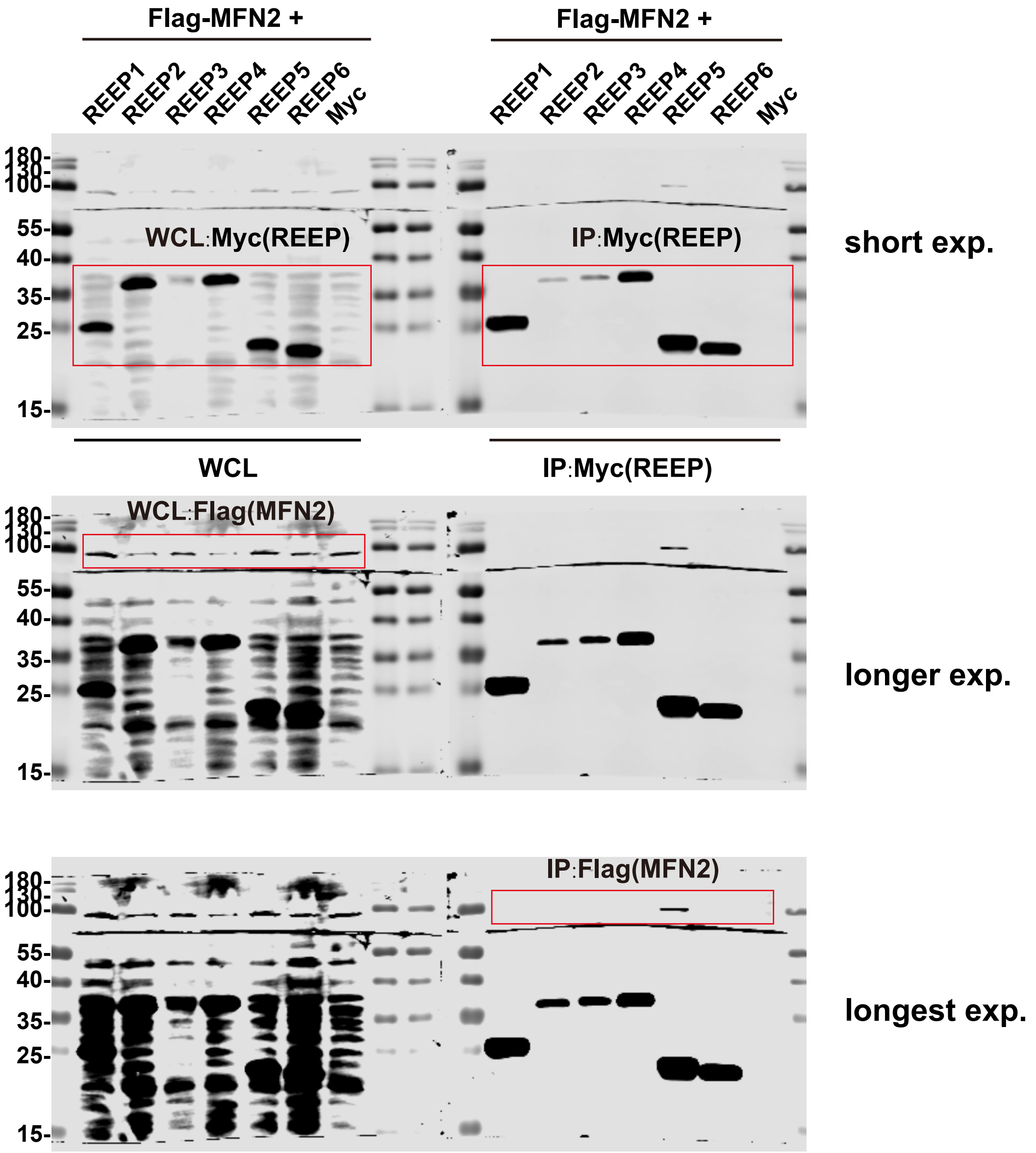

Fig. 1C

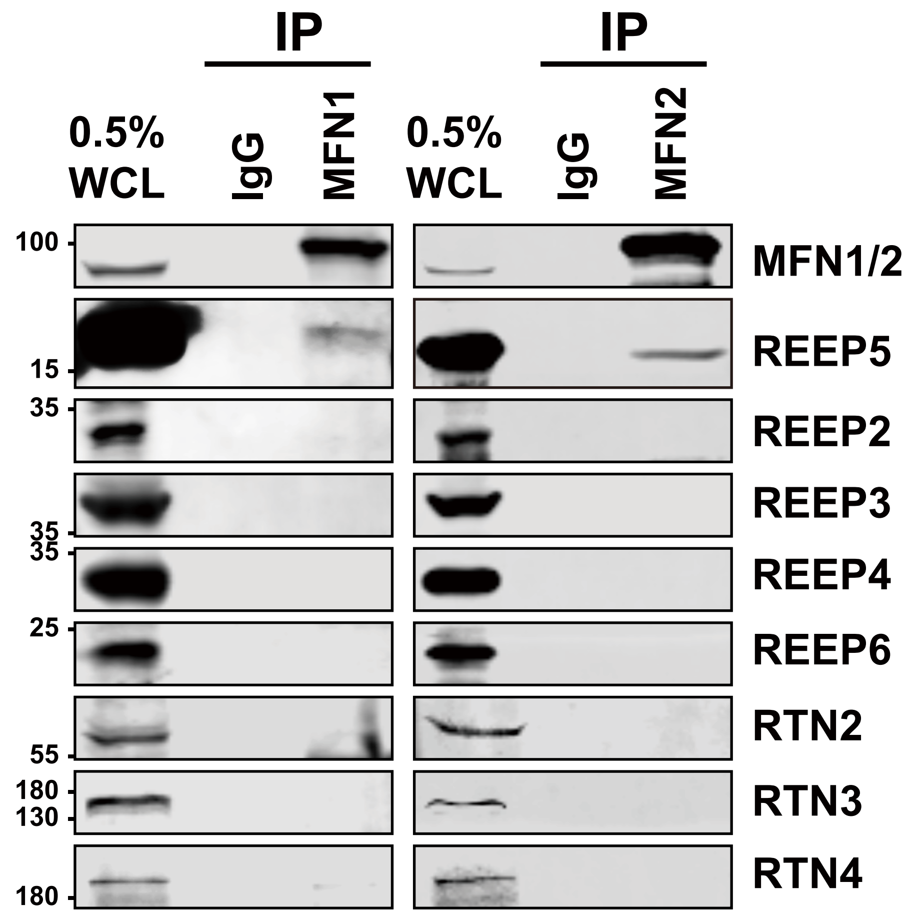
IP
IP
0.5%
WCL
IgG
MFN1
0.5%
WCL
IgG
MFN2
100
MFN1/2
15
REEP5
35
REEP2
35
REEP3
35
REEP4
25
REEP6
55
RTN2
180
130
RTN3
180
RTN4

Source data for Fig. 1C MFN1 IP

Orginal image files from which Fig. 1C was assembled.

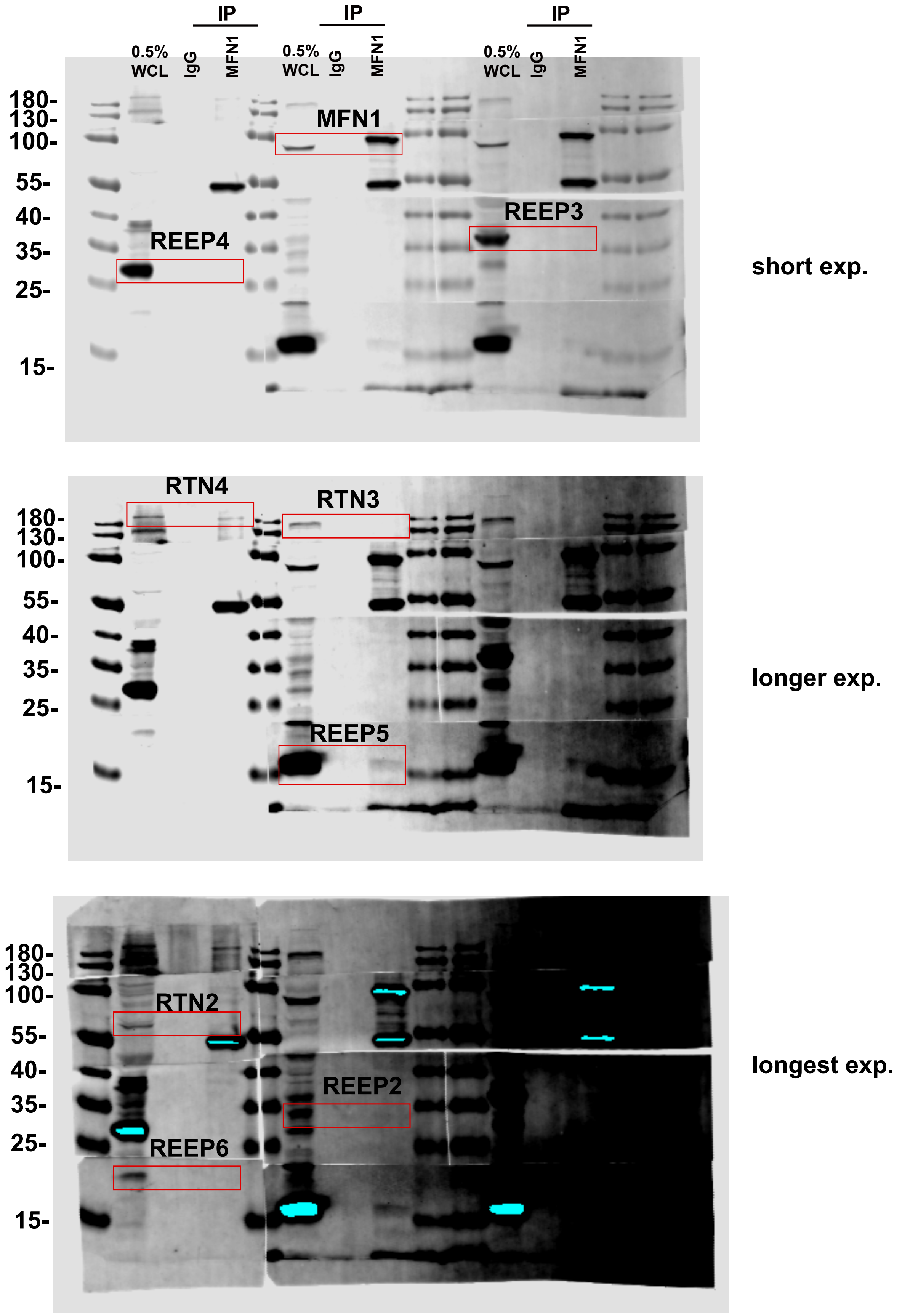

*Blue bands are automated markings for signal saturation by the Odyssey Imager.

Source data for Fig. 1C MFN2 IP

Orginal image files from which Fig. 1C was assembled.

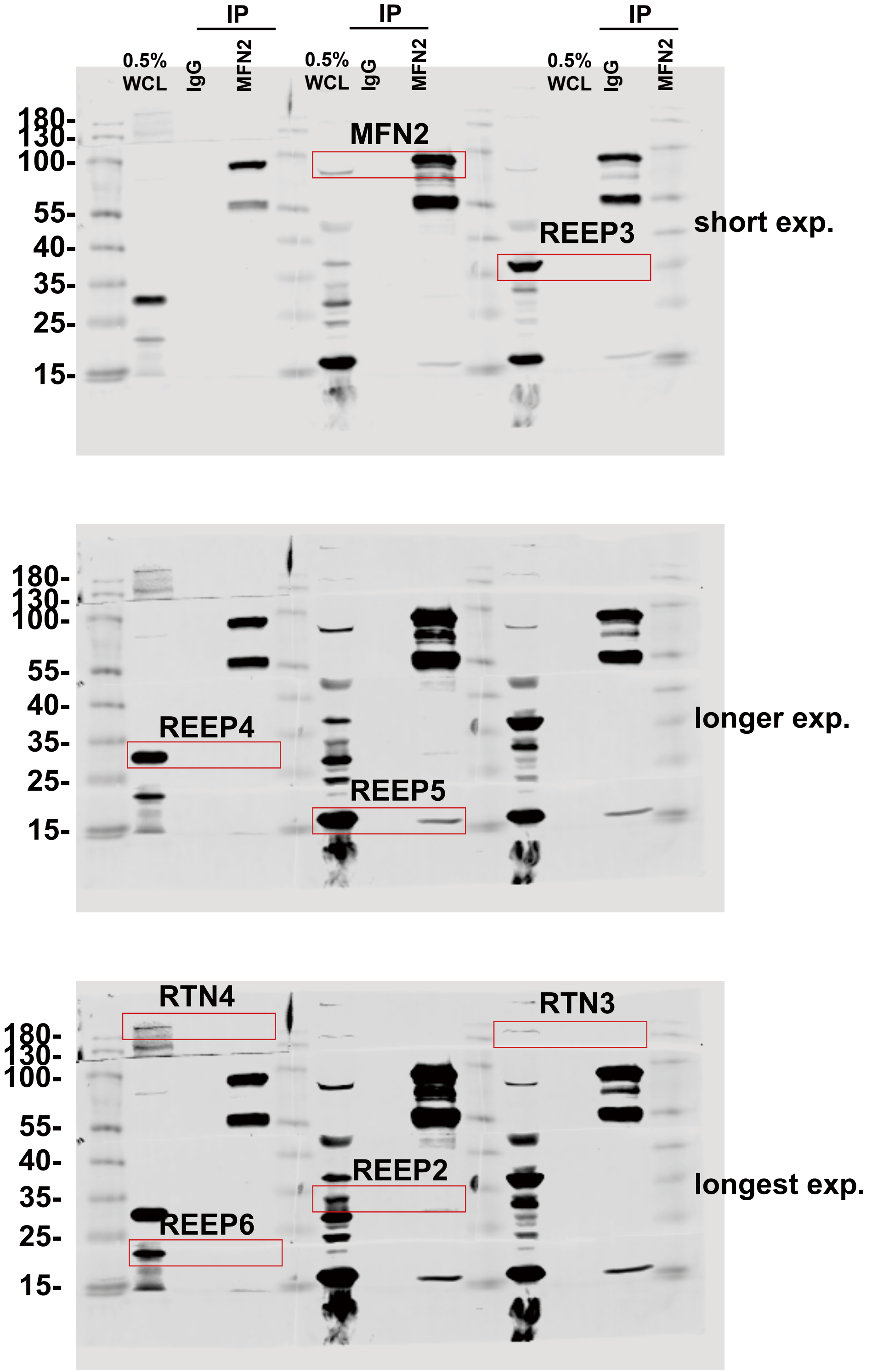

Top: probed with RTN2

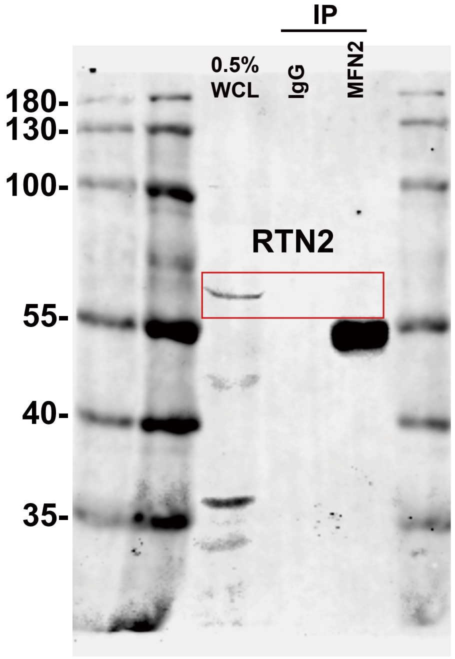

Bottom: probed with MFN2

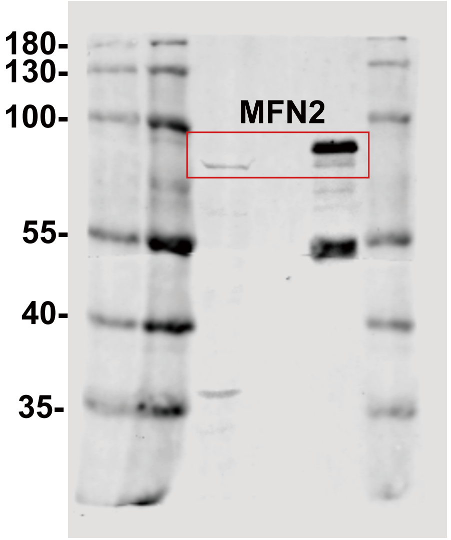

Fig. 1D

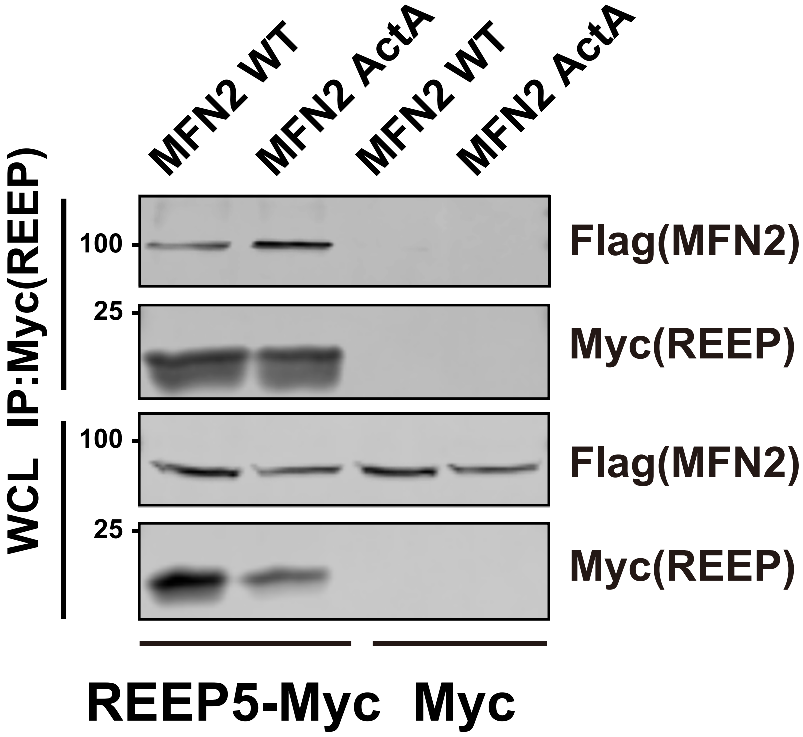
MFN2 WT
MFN2 ActA
MFN2 WT
MFN2 ActA
IP:Myc(REEP)
WCL
100
25
100
25
Flag(MFN2)
Myc(REEP)
Flag(MFN2)
Myc(REEP)
REEP5-Myc
Myc

Source data for Fig. 1D

Orginal image files from which Fig. 1D was assembled.

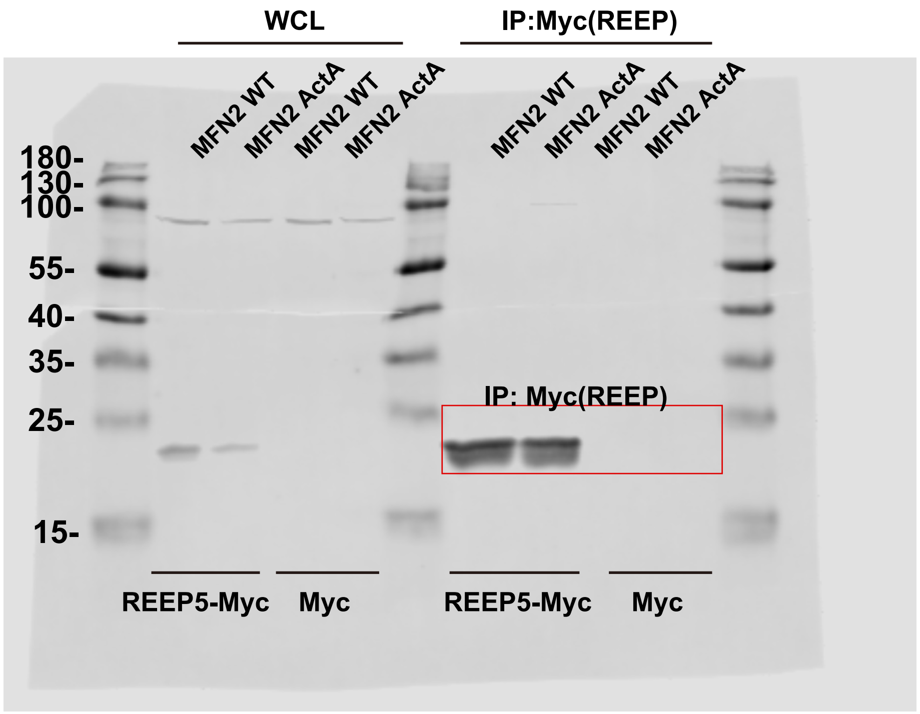

short exp.

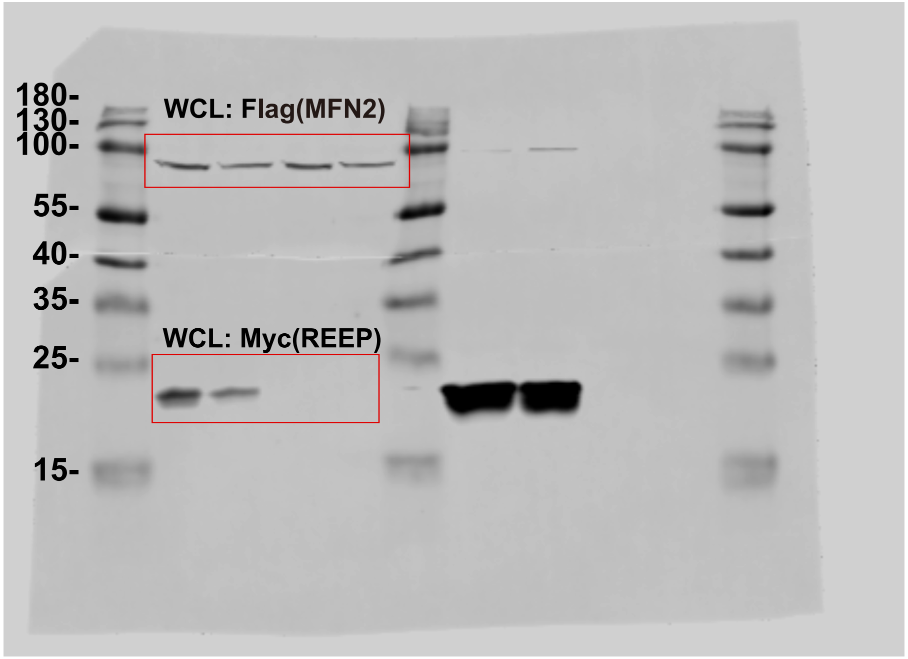

longer exp.

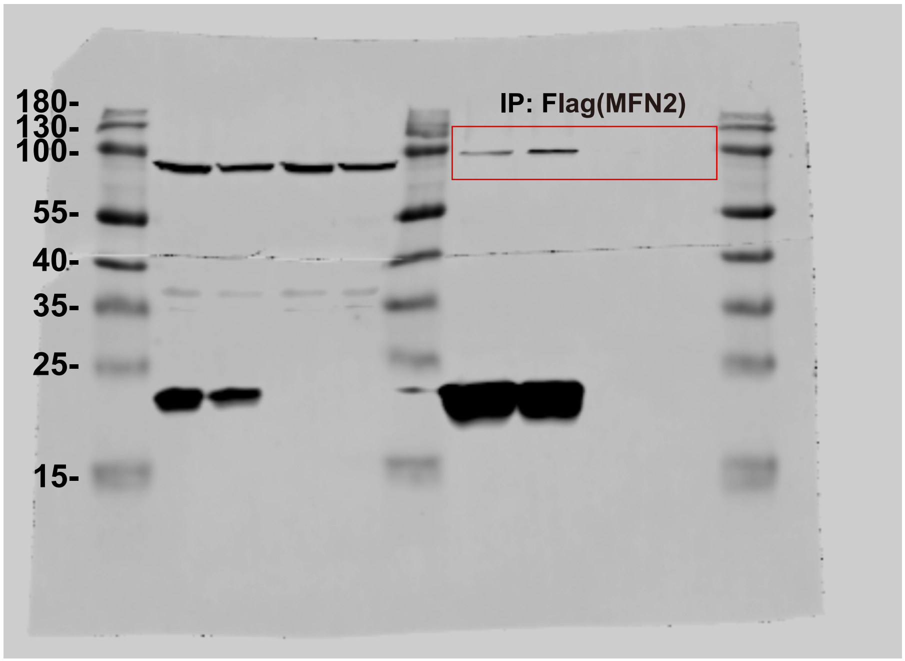

longest exp.

Fig. 1F

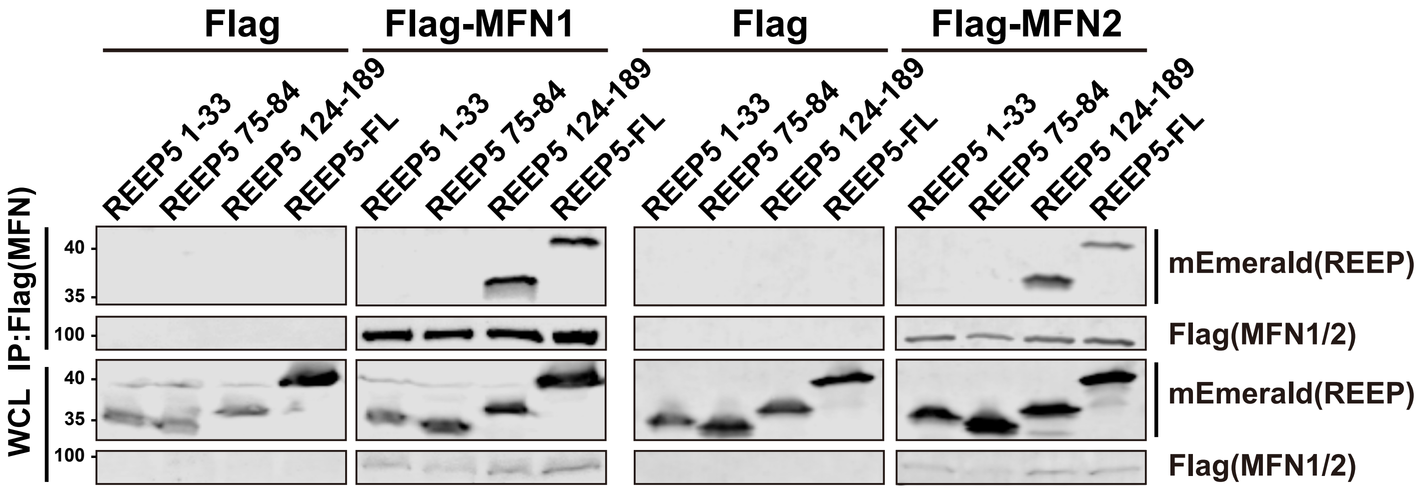

Flag
Flag-MFN1
Flag
Flag-MFN2
REEP5 1-33
REEP5 75-84
REEP5 124-189
REEP5-FL
IP:Flag(MFN)
WCL
40
35
100
mEmerald(REEP)
Flag(MFN1/2)

Source data for Fig. 1F

Orginal image files from which Fig. 1F was assembled.

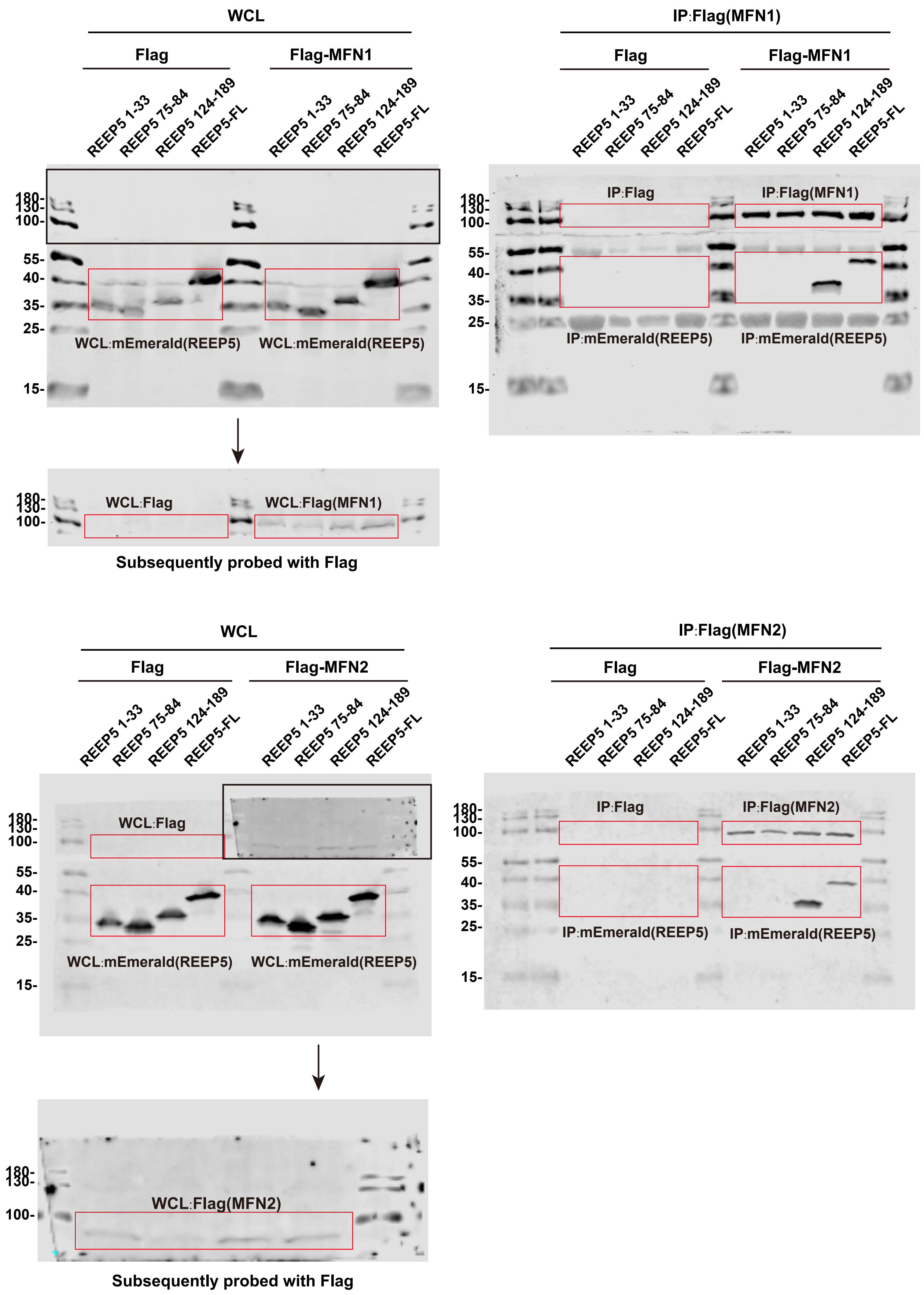

Fig. 1G

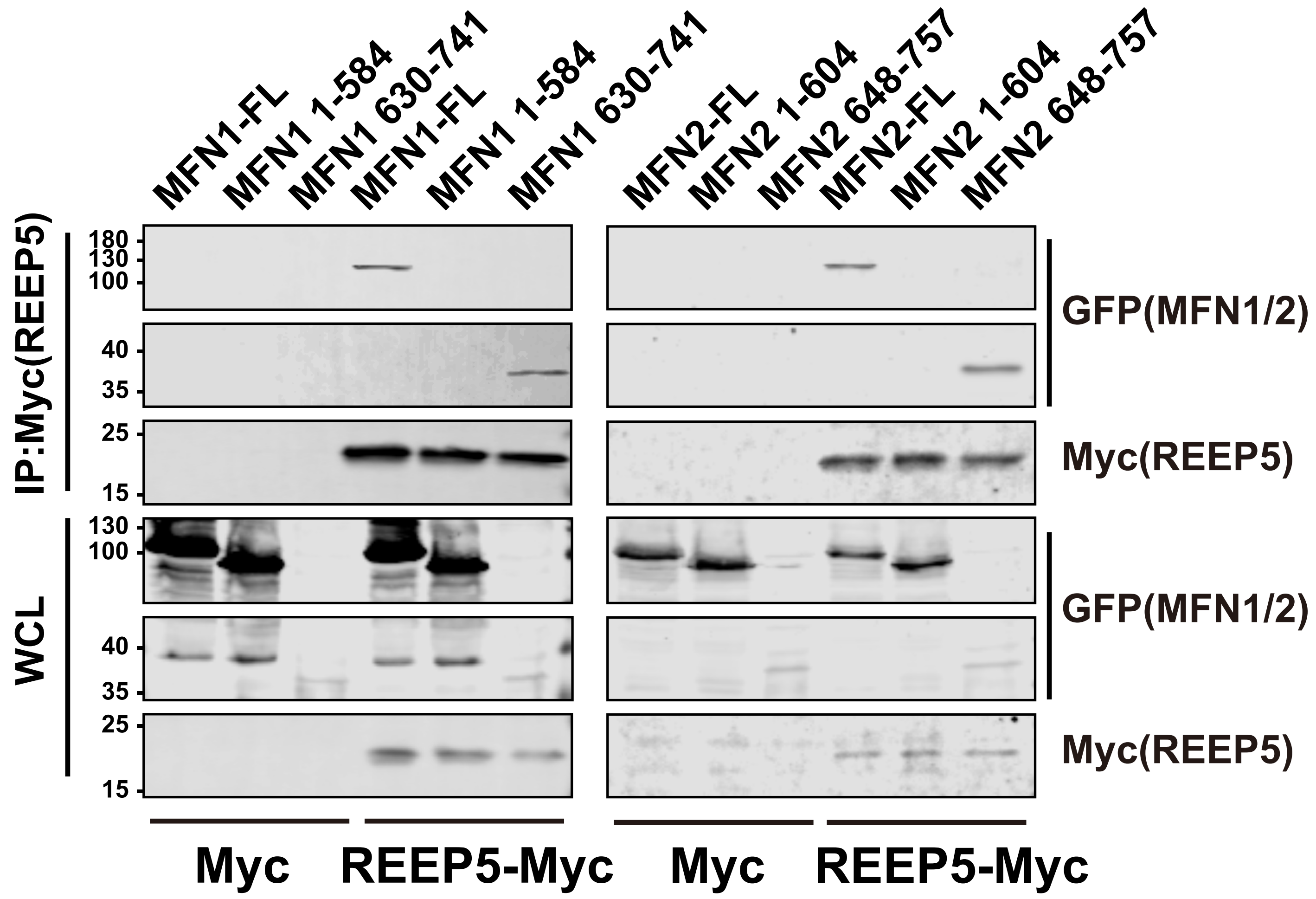
MFN1-FL
MFN1 1-584
MFN1 630-741
MFN1-FL
MFN1 1-584
MFN1 630-741
MFN2-FL
MFN2 1-604
MFN2 648-757
MFN2-FL
MFN2 1-604
MFN2 648-757
IP:Myc(REEP5)
WCL
180
130
100
40
35
25
15
130
100
40
35
25
15
GFP(MFN1/2)
Myc(REEP5)
GFP(MFN1/2)
Myc(REEP5)
Myc
REEP5-Myc
Myc
REEP5-Myc

Source data for Fig. 1G

Orginal image files from which Fig. 1G was assembled.

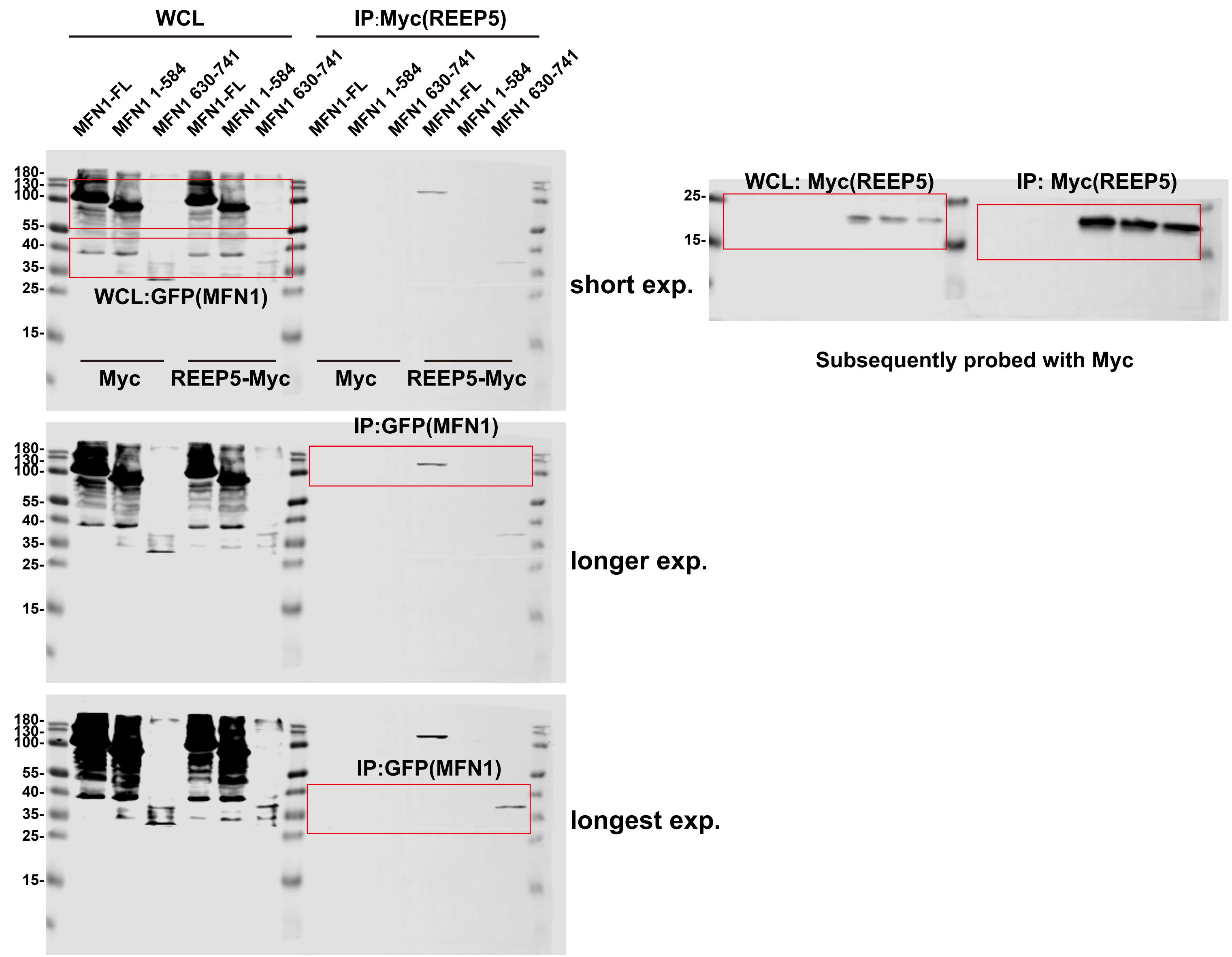

Orginal image files from which Fig. 1G was assembled.

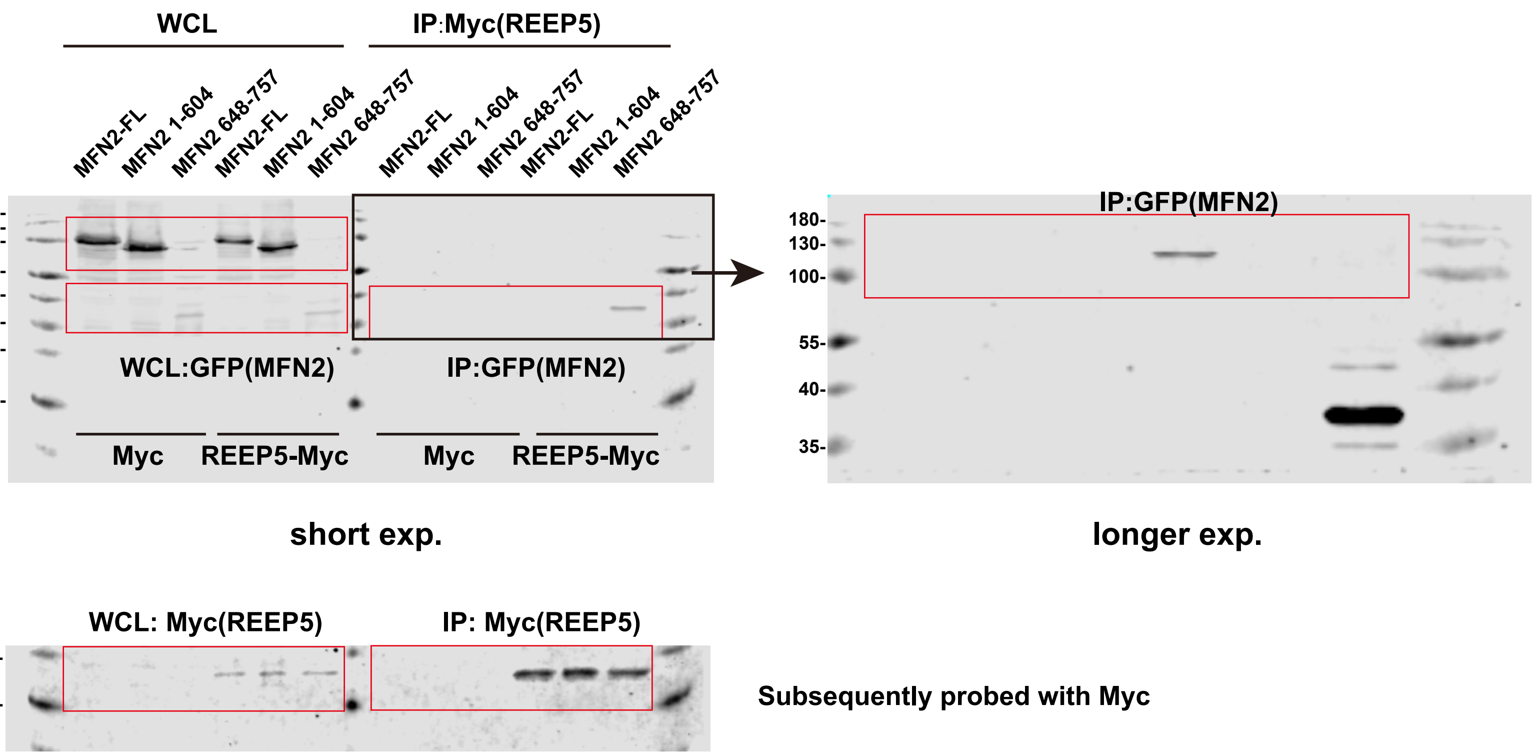

Fig. 1H

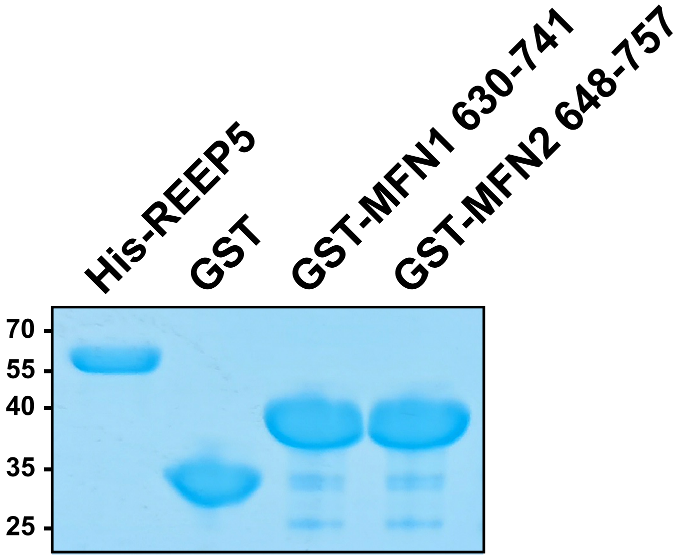

Purified proteins

Source data for Fig. 1H

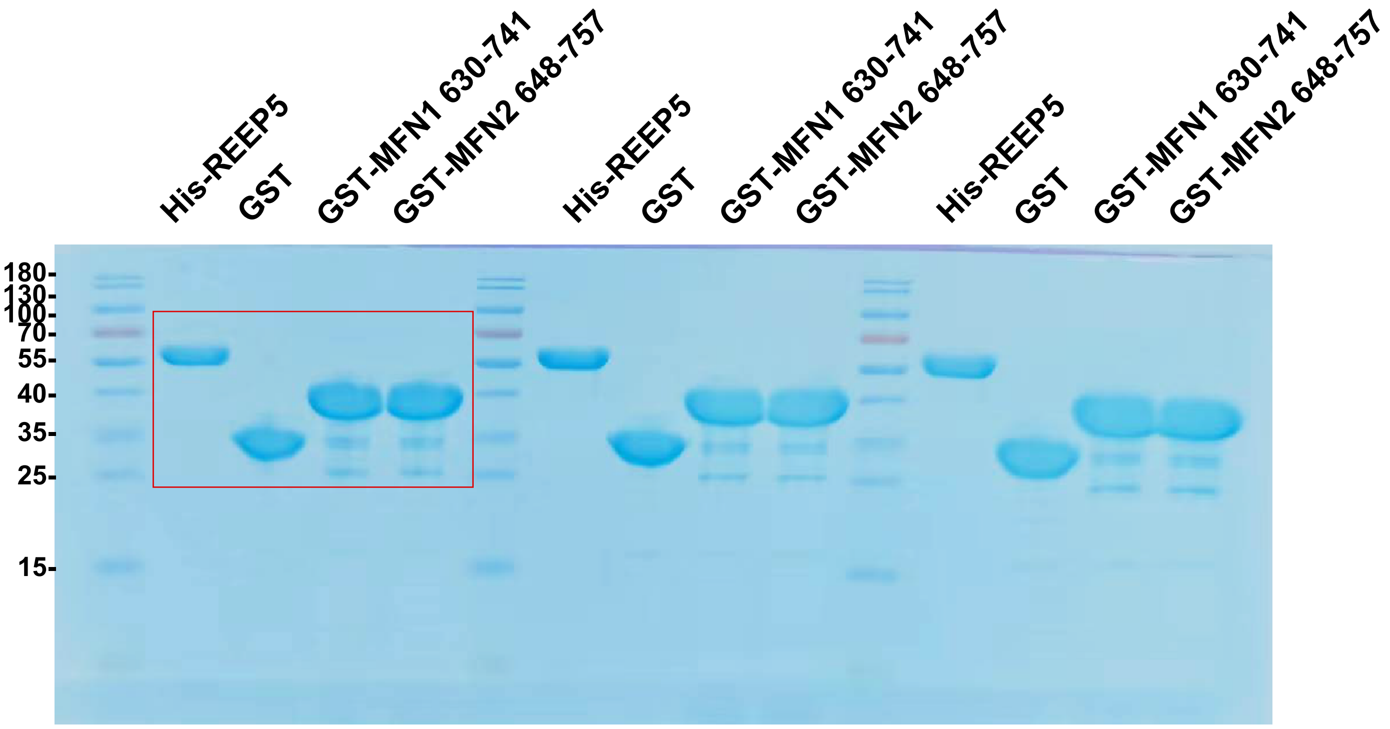

Fig. 1I

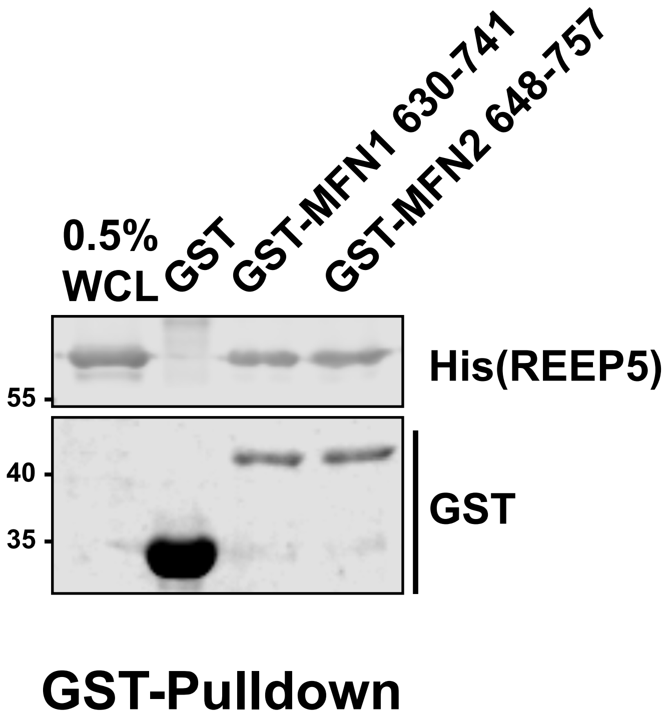
0.5%
WCL
GST
GST-MFN1 630-741
GST-MFN2 648-757
His(REEP5)
55
40
35
GST
GST-Pulldown

Source data for Fig. 1I

Orginal image files from which Fig. 1I was assembled.

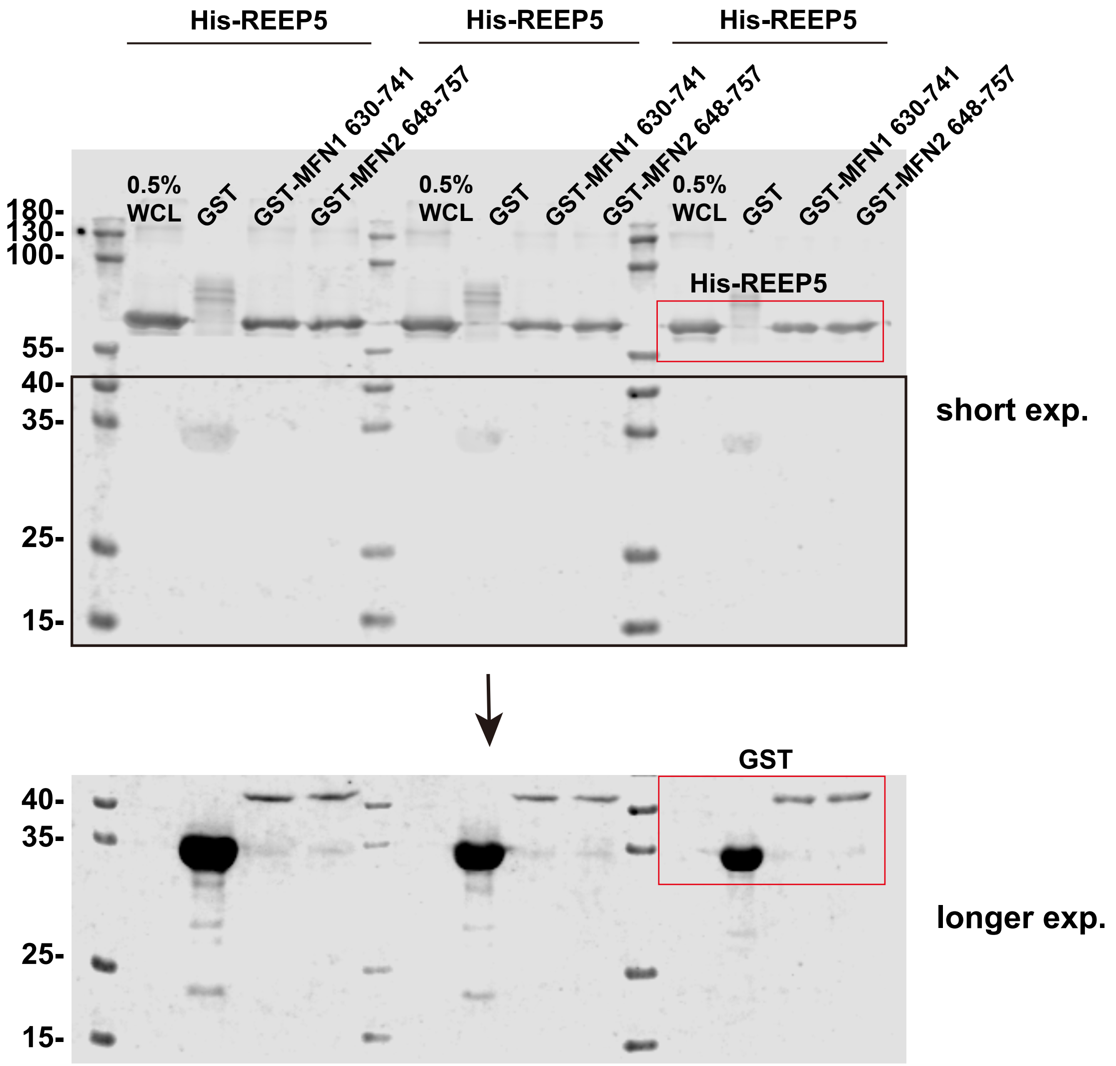

Supplement: SourceData F1 — is the source file for Fig. 1. [file JCB_202304031_SourceDataF1.pdf]
