# Supplementary material for: Dynamic interaction of REEP5–MFN1/2 enables mitochondrial hitchhiking on tubular ER
Source: J Cell Biol. 2024 Aug 12;223(10):e202304031. doi: 10.1083/jcb.202304031 (PMC11318672; doi:10.1083/jcb.202304031)

Supplemental Fig. 1A

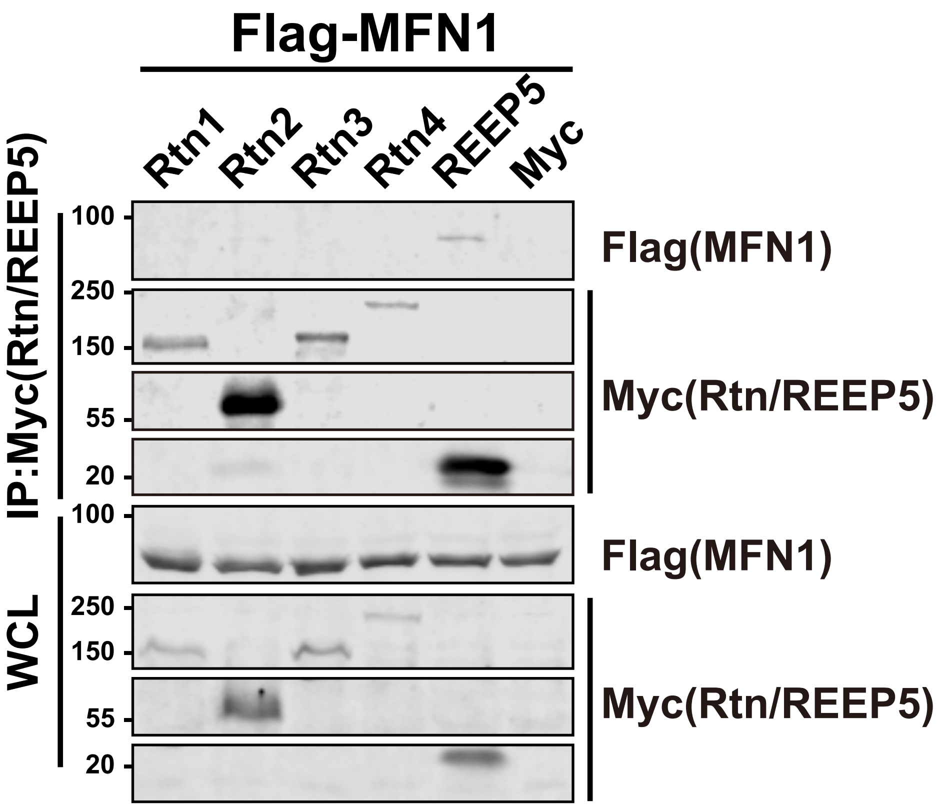

Source data for supplementary Fig. 1A

## Orginal image files from which Fig. S1A was assembled.

**Probed with Flag**

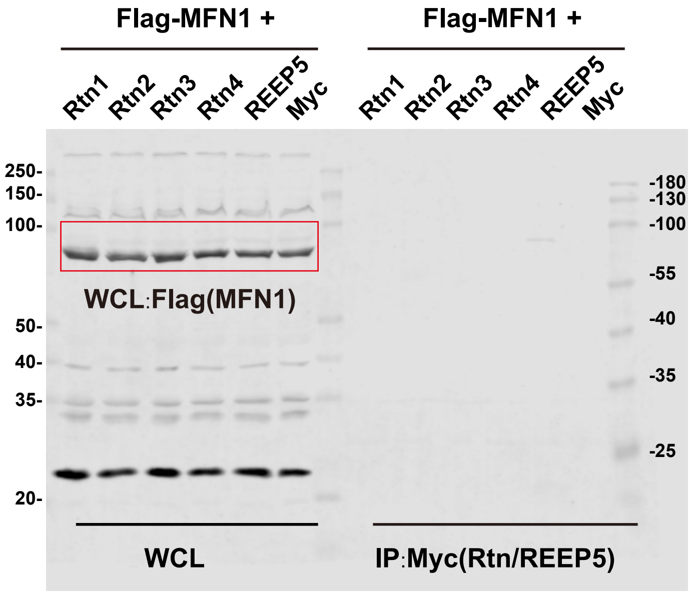


short exp.

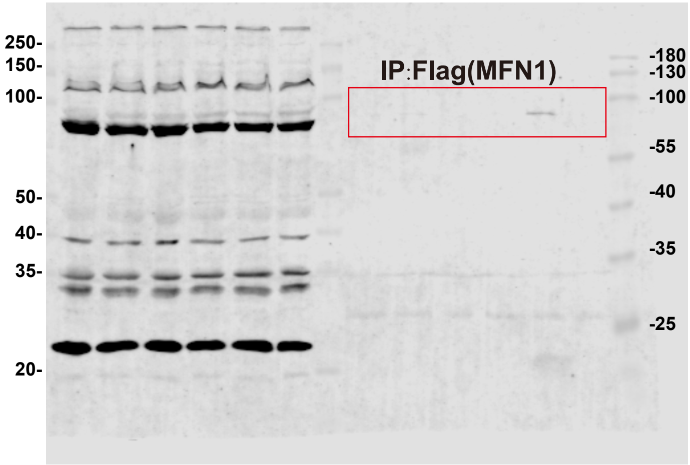


longer exp.

**Probed with Myc**

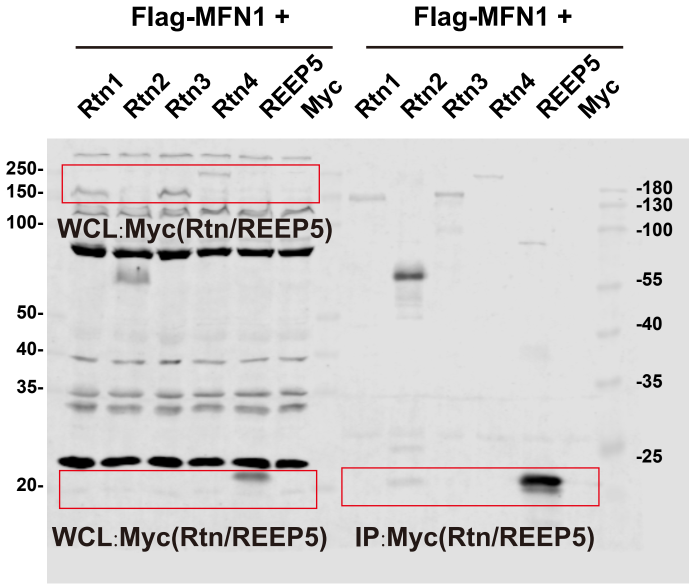


short exp.

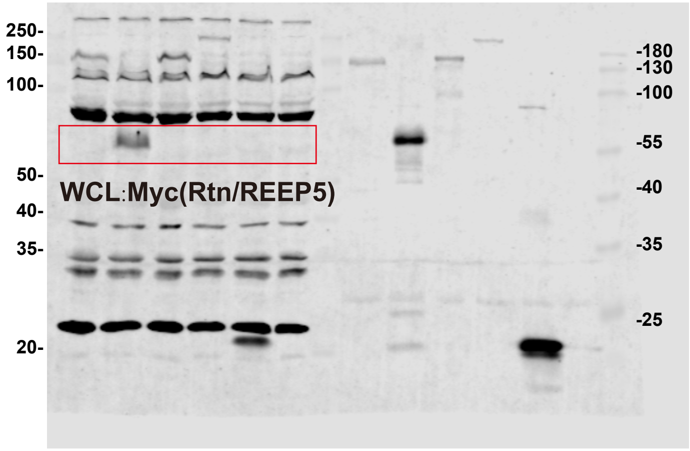


longer exp.

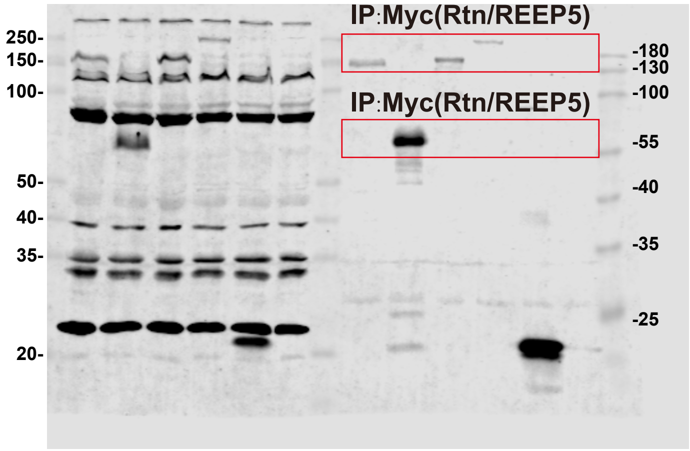


longest exp.

Supplemental Fig. 1B

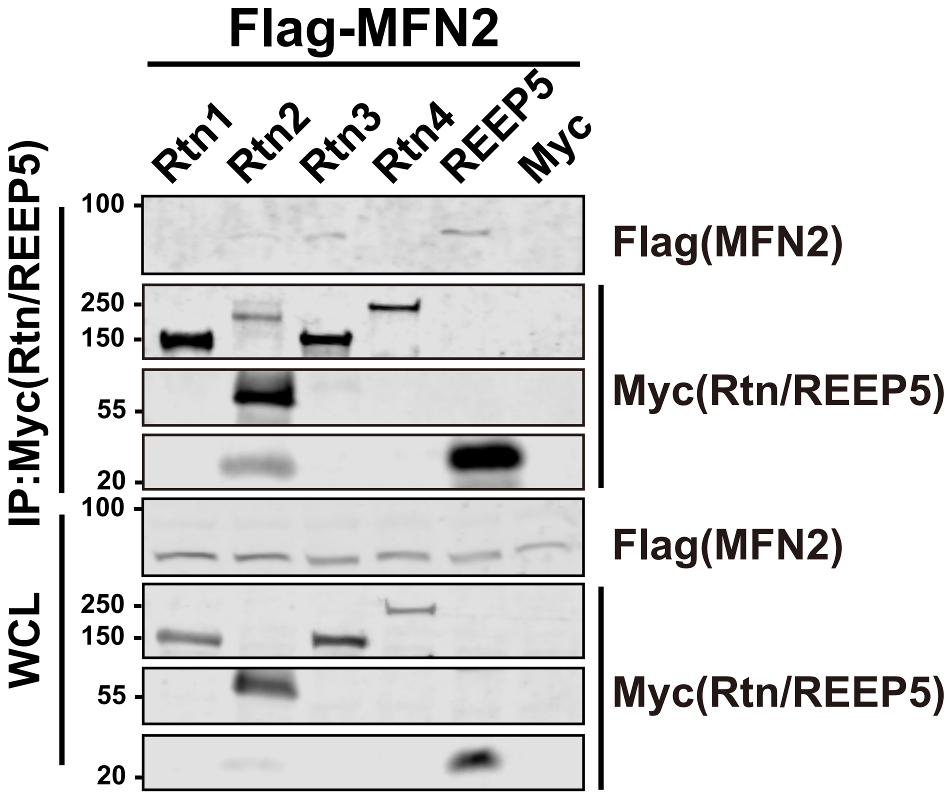

Source data for supplementary Fig. 1B

## Orginal image files from which Fig. S1B was assembled.

**Probed with Flag**

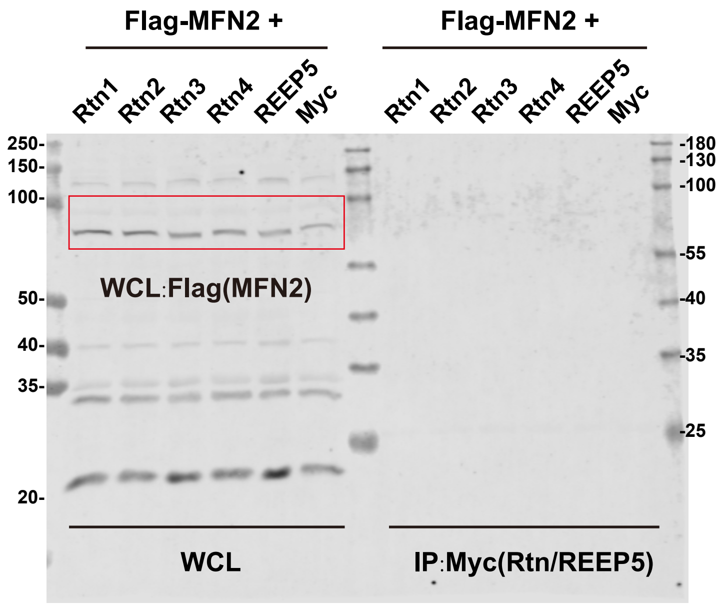


short exp.

**Probed with Myc**

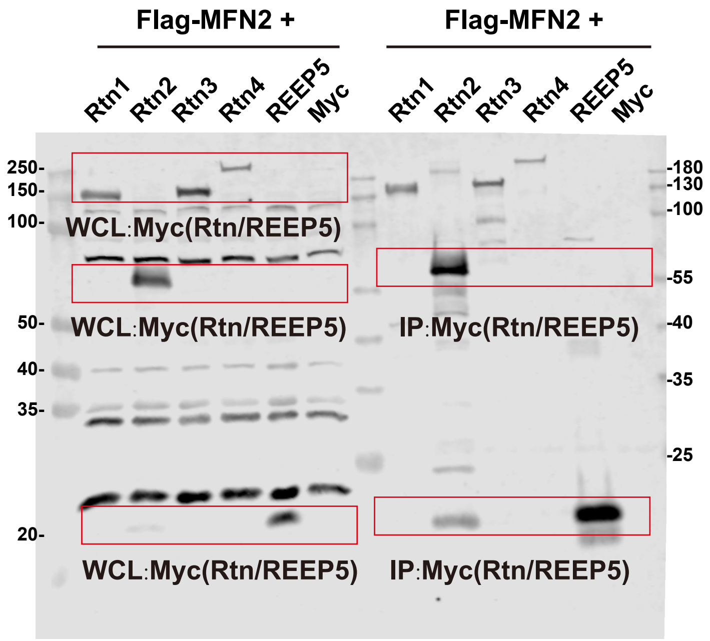


short exp.

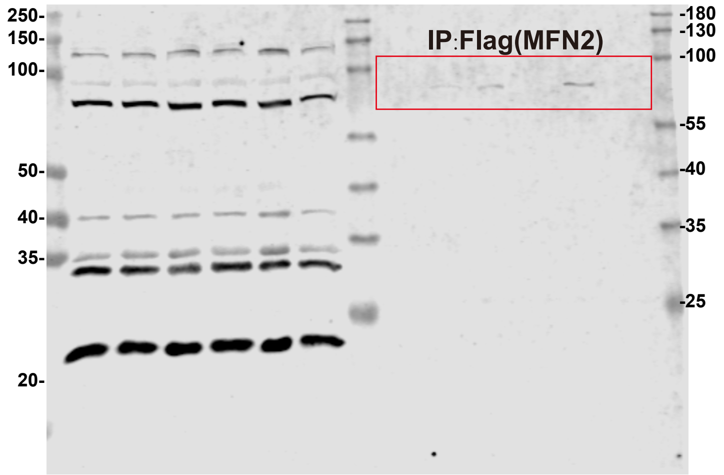


longer exp.

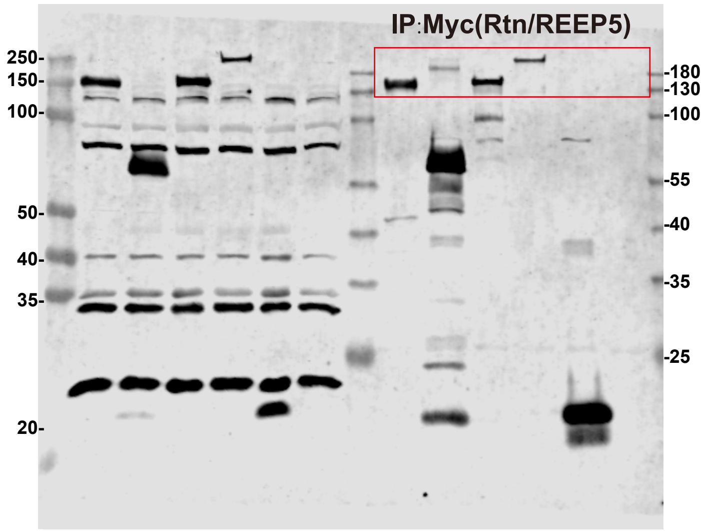


longer exp.

Supplemental Fig. 1C

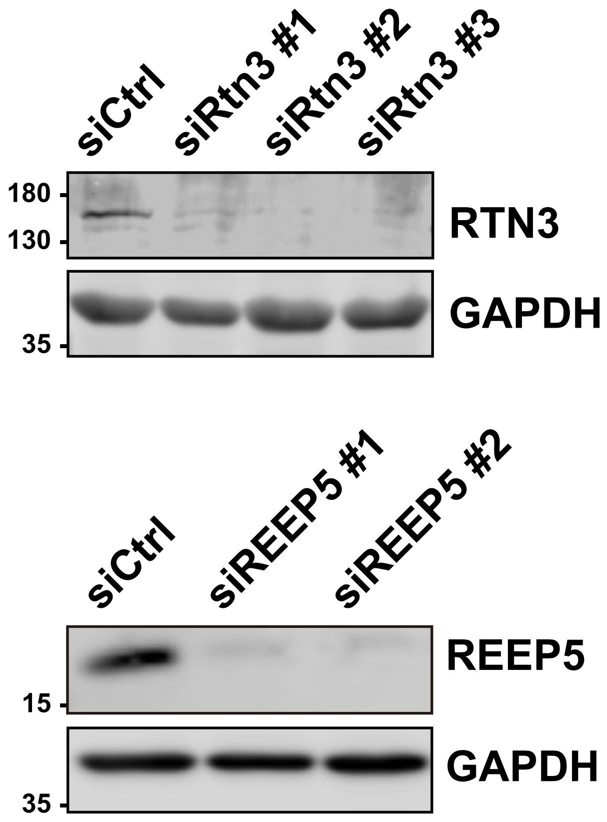

Source data for supplementary Fig. 1C

## Orginal image files from which Fig. S1C was assembled.

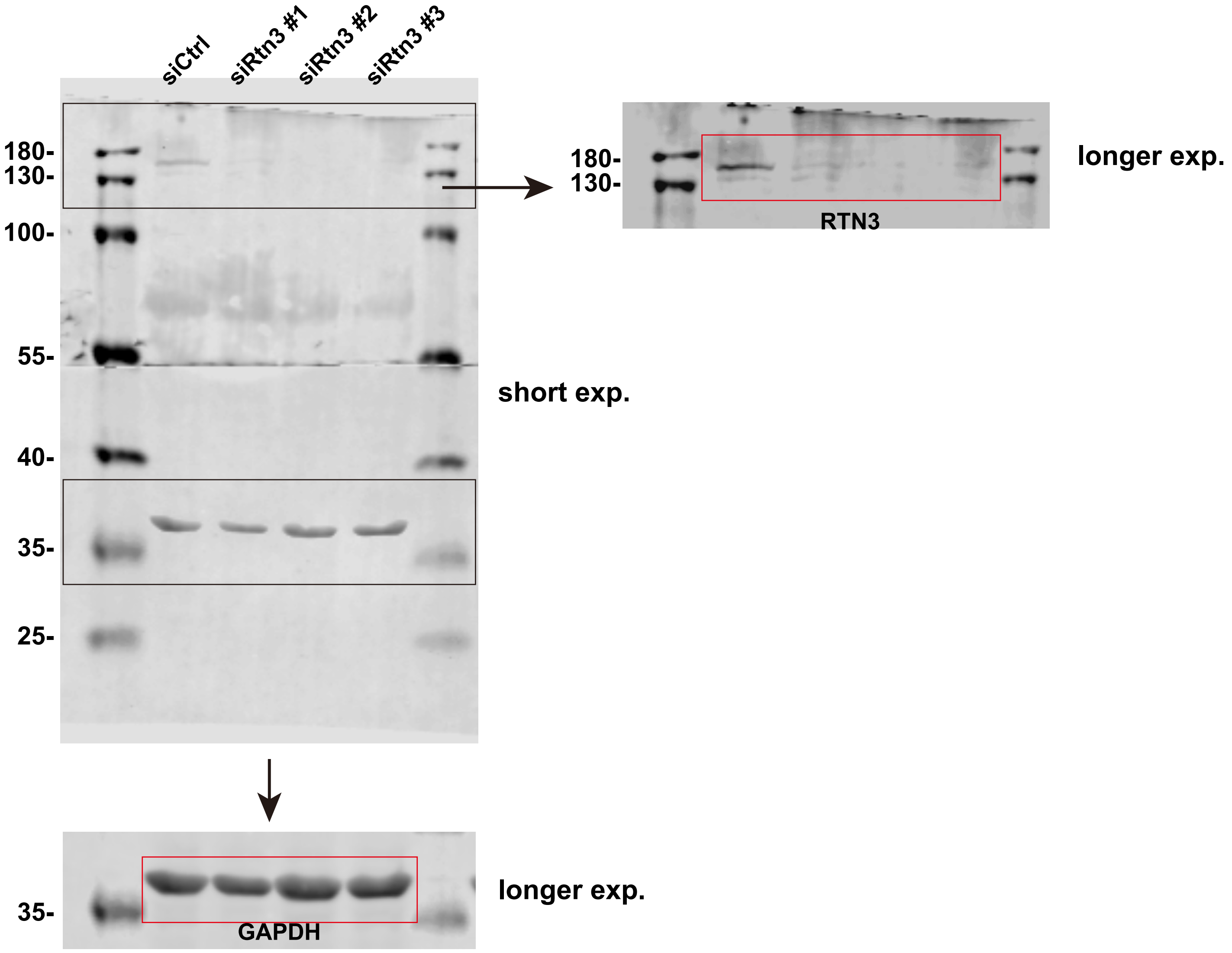


## Orginal image files from which Fig. S1C was assembled.

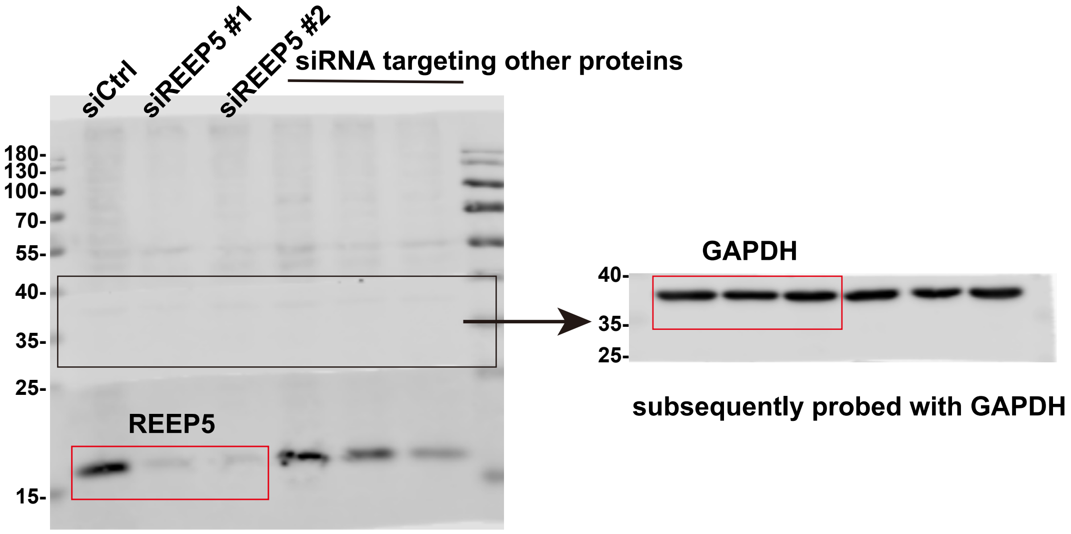

Supplemental Fig. 1D

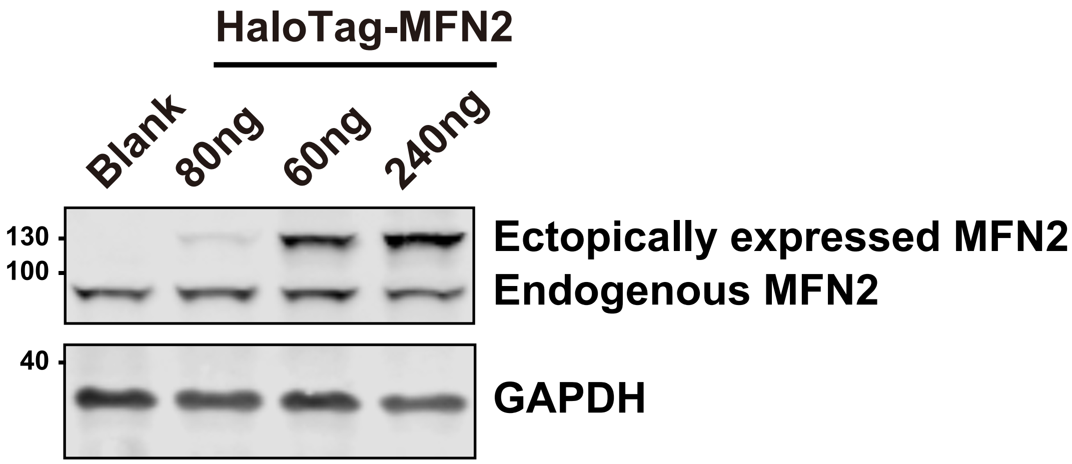

Source data for supplementary Fig. 1D

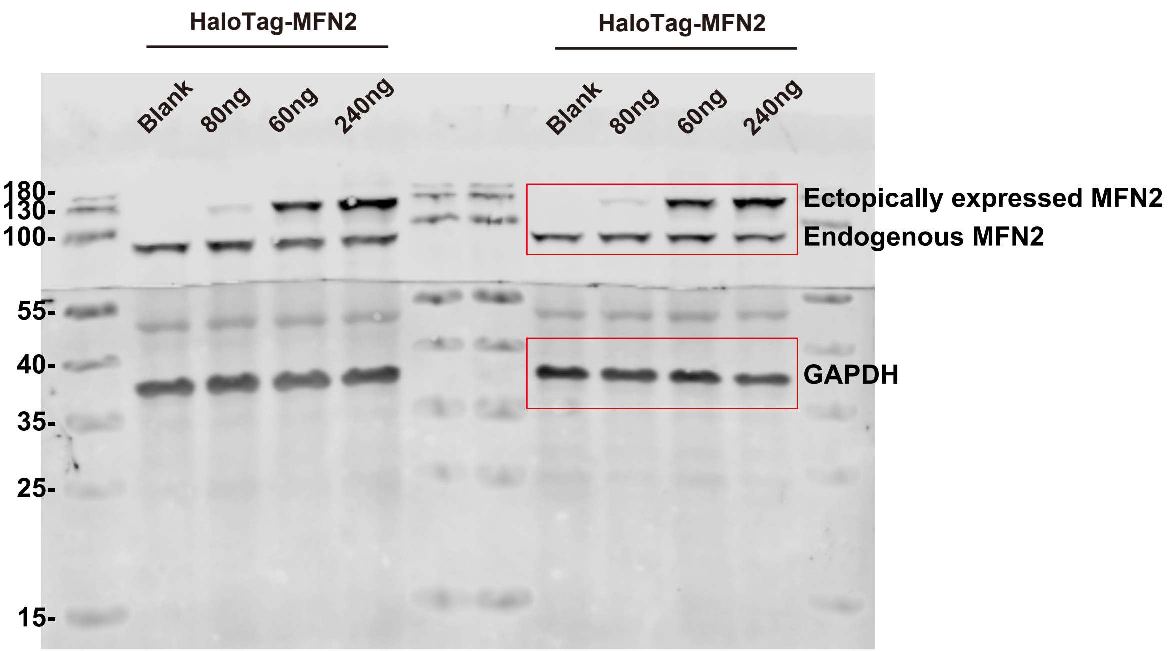

Supplement: SourceData FS1 — is the source file for Fig. S1. [file JCB_202304031_SourceDataFS1.pdf]
