# Supplementary figures and images for: Dynamic interaction of REEP5–MFN1/2 enables mitochondrial hitchhiking on tubular ER
Source: J Cell Biol. 2024 Aug 12;223(10):e202304031. doi: 10.1083/jcb.202304031 (PMC11318672; doi:10.1083/jcb.202304031)

Supplemental Fig. 2C

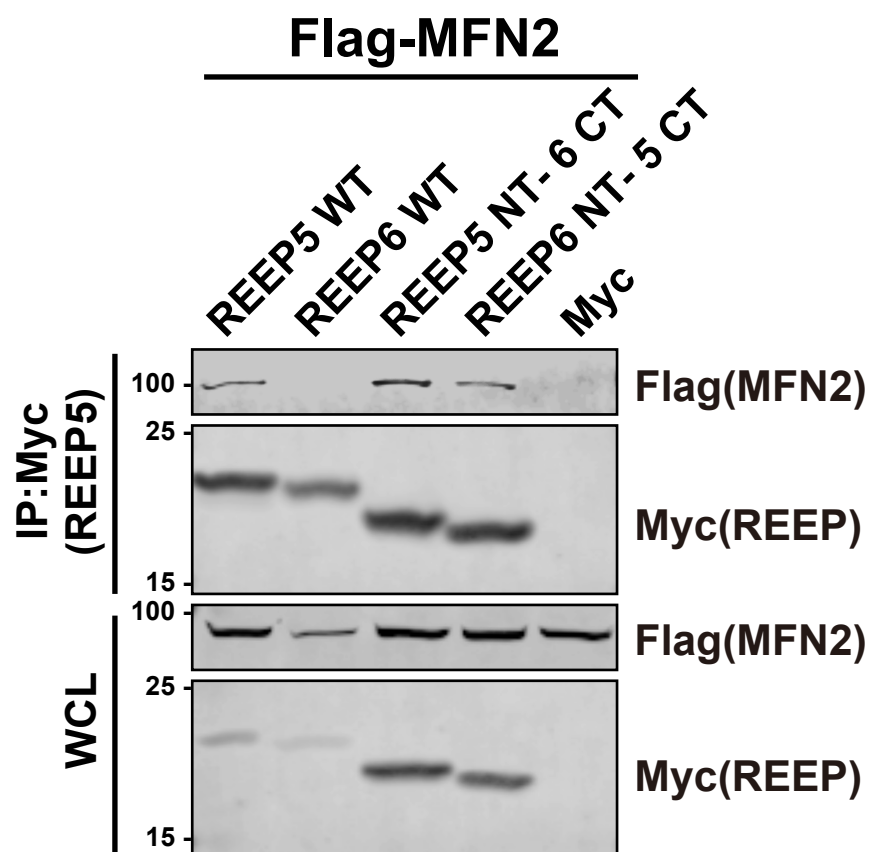

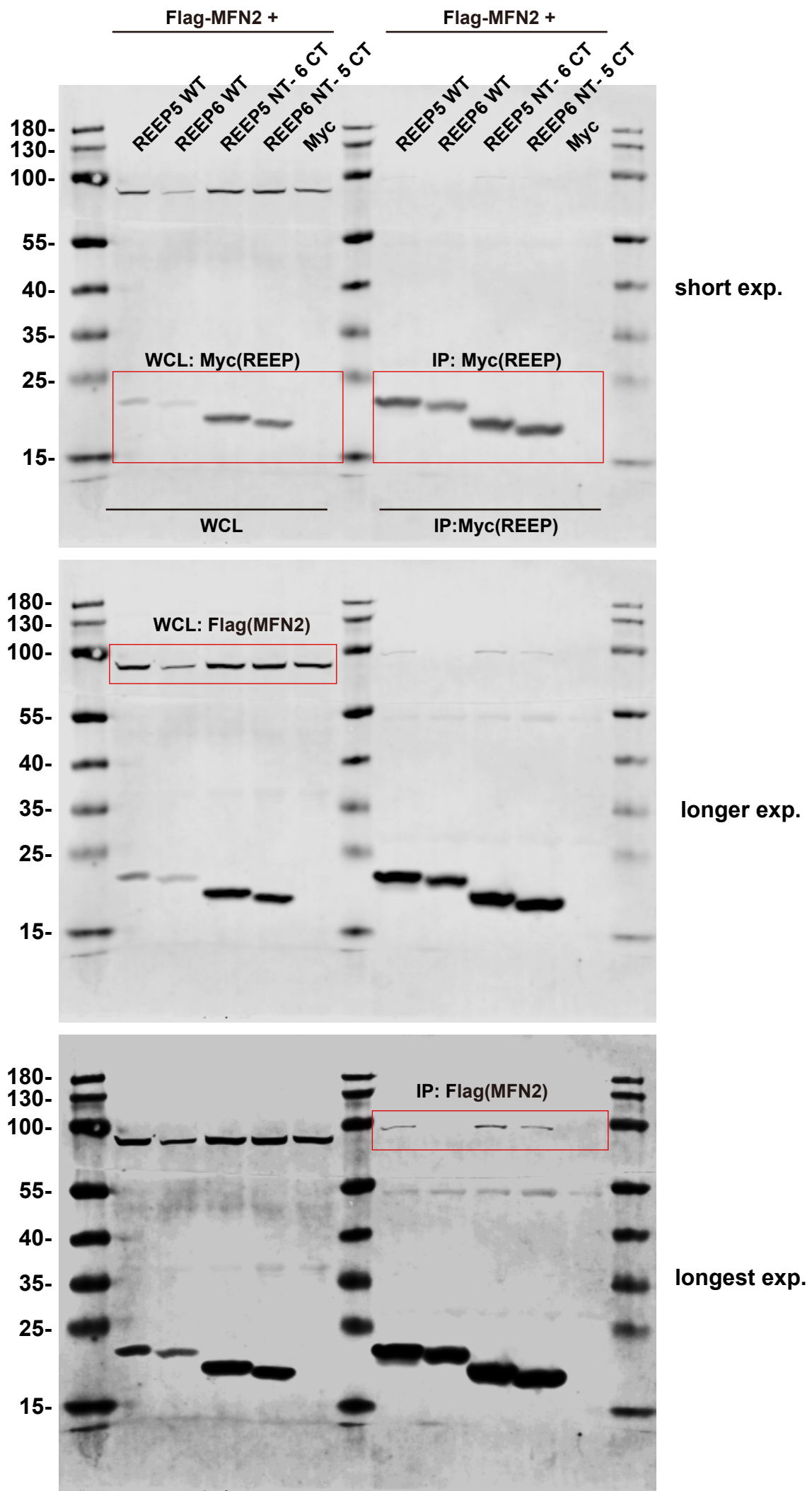

Supplemental Fig. 2E

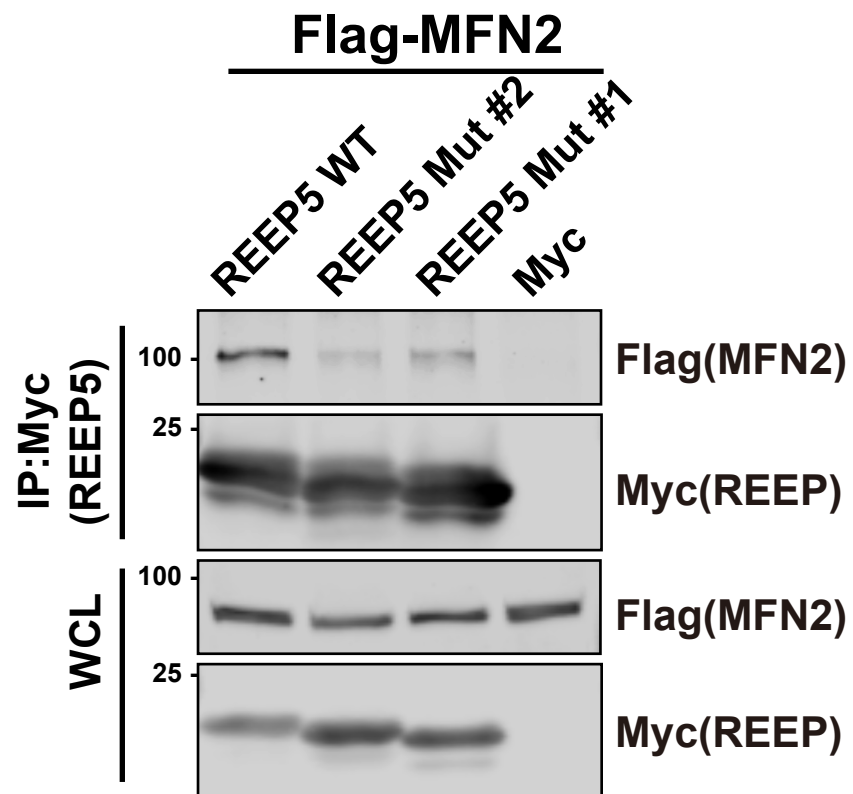

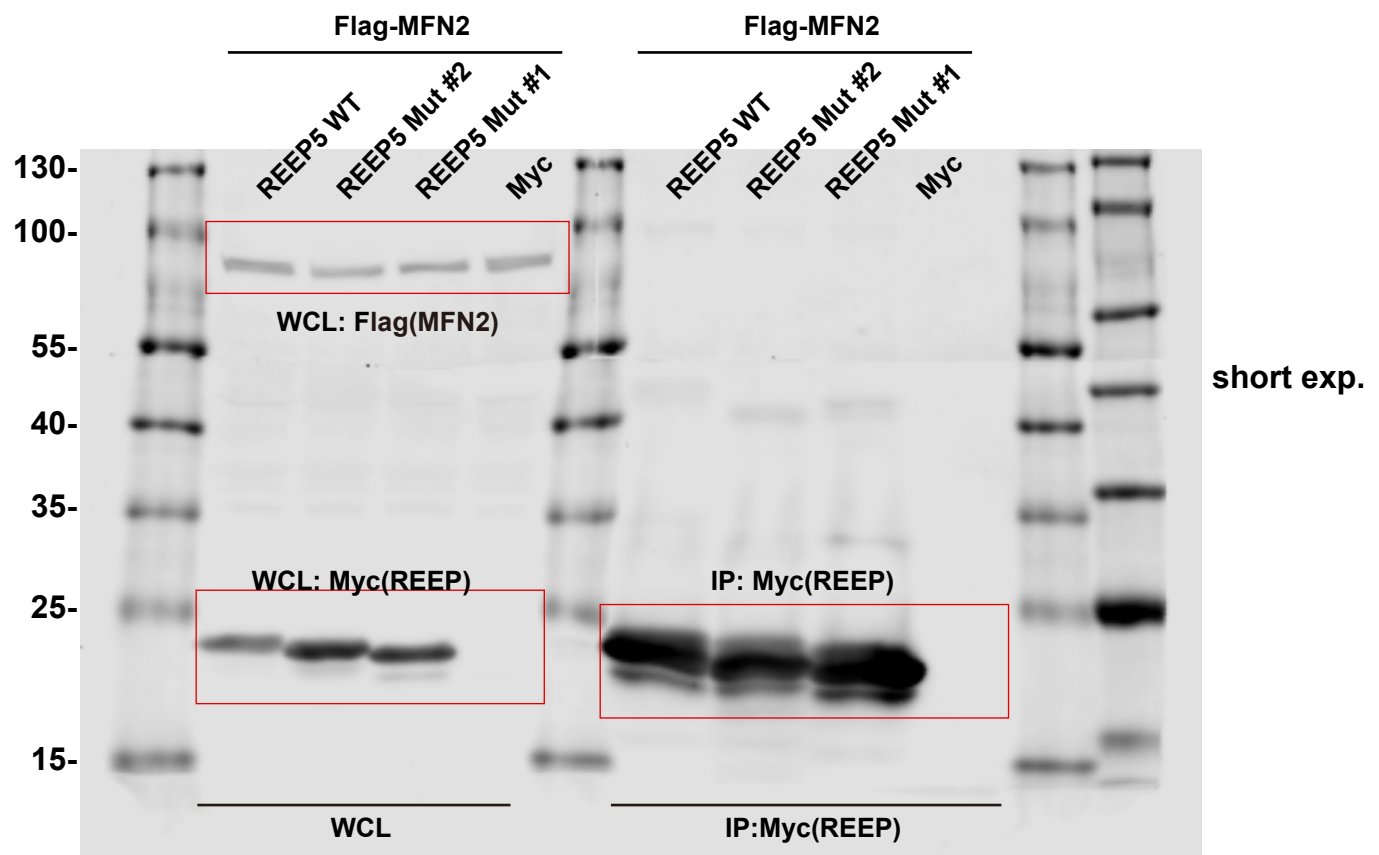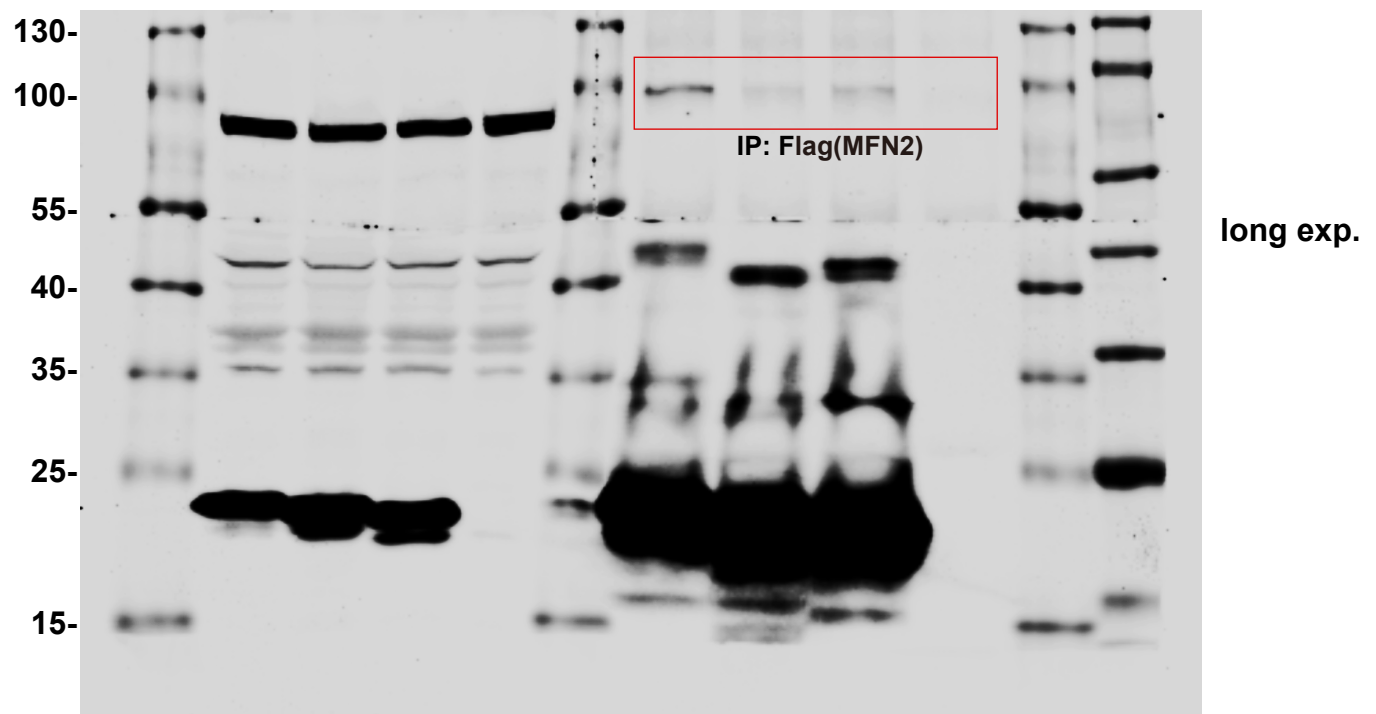

Supplemental Fig. 2G

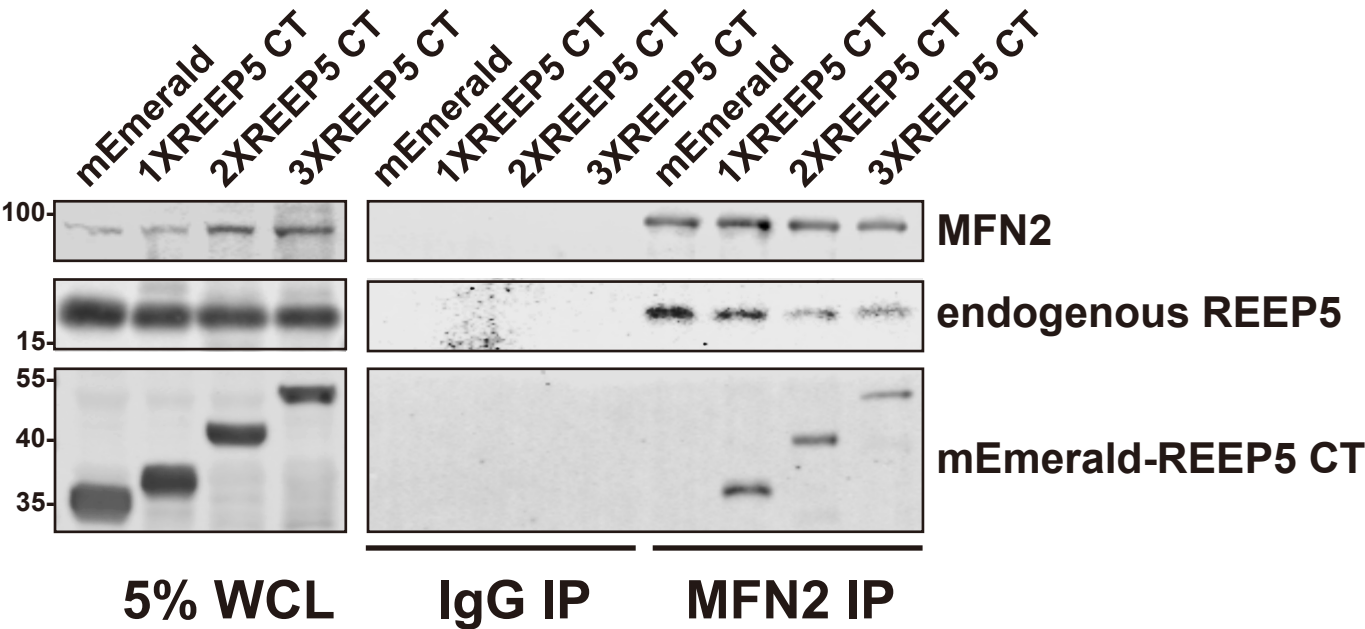

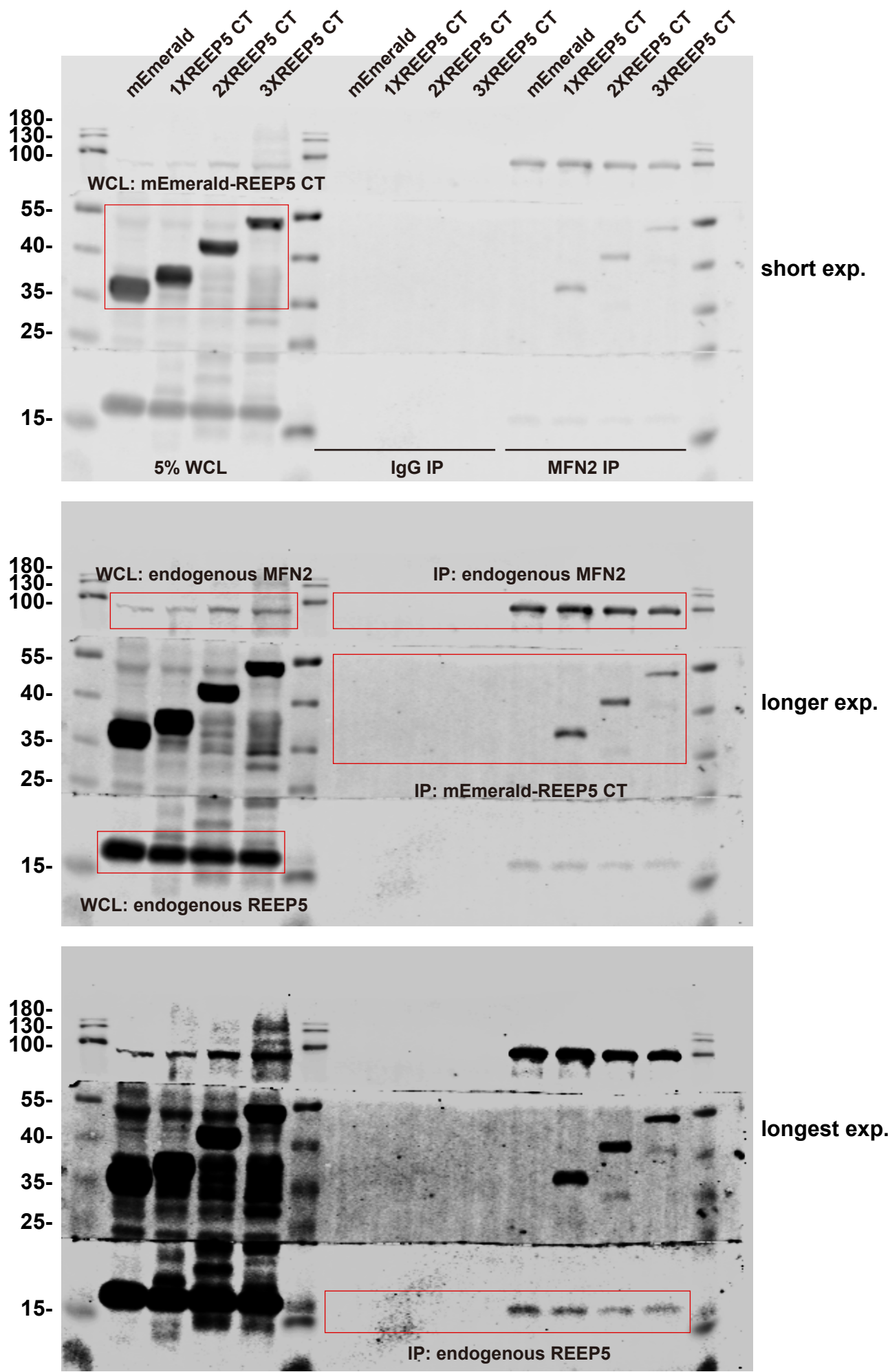

Supplement: SourceData FS2 — is the source file for Fig. S2. [file JCB_202304031_SourceDataFS2.pdf]
